# OXTR-mediated signaling in astrocytes contributes to anxiolysis

**DOI:** 10.1038/s41380-024-02870-5

**Published:** 2024-12-19

**Authors:** Carl-Philipp Meinung, Laura Boi, Sareh Pandamooz, David Mazaud, Grégory Ghézali, Nathalie Rouach, Inga D. Neumann

**Affiliations:** 1 Department of Behavioral and Molecular Neurobiology, University of Regensburg, Regensburg, Germany; 2 Stem Cells Technology Research Center, Shiraz University of Medical Sciences, Shiraz, Iran; 3 Center for Interdisciplinary Research in Biology, Collège de France, CNRS, INSERM, Université PSL, Labex Memolife, Paris, France

**Keywords:** Neuroscience, Molecular biology

## Abstract

Astrocytes are an indispensable part of signal processing within the mammalian brain. Thus, the mode of action of a neuropeptide such as oxytocin (OXT) can only be fully understood considering this integral part of the CNS. Here, we show that OXT regulates astrocytic gene expression, intracellular signaling and specific proteins both in vitro and in vivo. This translates into rapid regulation of astroglial structural and functional properties including cytoskeletal plasticity, coverage of synapses and gap-junction coupling. At the molecular level, we identify the previously undescribed Sp1-Gem signaling cascade as the key driver for these cell type-specific OXT effects. Finally at the behavioral level, we found in vivo that OXT requires astrocytes to exert its well described anxiolytic properties within the hypothalamic paraventricular nucleus. Thus, our study points to OXT receptor-expressing astrocytes as a critical component of the brain OXT system.

## Introduction

Due to its multiple physiological and behavioral functions, there has been a growing scientific interest in the nonapeptide oxytocin (OXT) and its cognate receptor (OXTR) over the last decades. The brain OXT system promotes various cognitive and social functions, and exerts robust anxiolytic and anti-stress effects (reviewed in refs. [1, 2]), and is, thus, discussed as a potential therapeutic target for stress-related psychopathologies [3–7]. In this context, a detailed understanding of the multiple modes of action of OXT at cellular level - both in neurons and astrocytes - is of high relevance. OXT effects are mainly mediated via the OXTR, a G protein-coupled receptor expressed in multiple cell types of the CNS, including astrocytes [8, 9]. In neurons, OXT increases intracellular calcium (Ca^2+^) either from intracellular stores via activation of the protein kinase C (PLC) pathway or via activation of Ca^2+^influx [10–13]. Further, Ca^2+^-dependent downstream mechanisms involve the activation of transcription factors via the mitogen-activated protein kinase (MAPK) signaling pathway and, thus, regulation of protein synthesis. Ca^2+^ influx and activation of the MAPK pathway in hypothalamic neurons are essential for the anxiolytic effects of OXT [1, 10, 13].

Although our understanding of the vital role of astrocytes in information processing within the brain and the regulation of behavior has expanded over the past decade [14, 15], the effects of OXT on astrocytic cells, specifically on OXTR-coupled signaling and its resulting cellular and behavioral consequences, are rather poorly understood. Astrocytic activation by OXTR triggers various intracellular signaling pathways, such as PLC, MAPK and protein kinase A (PKA), potentially leading to modifications in astrocytic morphology and function [16]. Pioneering studies observed a reversible retraction of glial processes from OXT neurons and, consequently, reduction in direct neuronal-glial juxtapositions and improved neuronal communication in the hypothalamus of lactating rat dams [17]. These morphological adaptations may support physiological adaptations of OXT neurons in lactation, which display high activity and synchronicity [18]. Thus, glial structural plasticity likely results in increased glutamate availability at the synaptic cleft [19], elevated glutamate spillover from uncovered to neighbouring synapses [20], and, consequently, a stronger depression of GABAergic transmission via activation of presynaptic mGluRs. However, whether these adaptations are due to direct actions of OXT on astrocytic cells, which express the OXTR in various brain regions [16], is unknown.

Over the past years several studies have revealed an unexpected role of astrocytes in the modulation of anxiety-like behavior, for example in the hypothalamic paraventricular nucleus (PVN), hippocampus and amygdala [21–23]. However, a potential involvement of astrocytic OXTR signaling in behavioral regulation is rather unknown [15]. Recently, OXTR-mediated modulation of the astro-neuronal network in the central amygdala has been shown to play a role in the anxiolytic effect of OXT in the context of chronic pain, as observed in an animal model of neuropathic pain [24].

Here, we studied OXTR-mediated signaling and strictural plasticity of astrocytes in a cell type-specific way. We show that OXT causes rapid remodeling of astrocytes and simultaneous impairment of astrocytic networks. We further demonstrate that OXT exerts these effects via the previously undescribed Sp1-Gem signaling pathway, which marks it as a critical component of the cell type-specific response of astroglial cells to OXT. Finally, by selectively downregulating astrocytic OXTR signaling in vivo, we reveal that OXT requires direct binding to astrocytes to bring about its anxiolytic effect within the PVN.

## Materials and methods

### Animals

Adult male Wistar rats (250–300 g; Charles River, Sulzfeld, Germany) were housed under standard laboratory conditions. After surgery, rats were single-housed for either five days (guide cannula implantations) or 3 weeks (viral microinfusions) before taking biological samples or behavioral testing.

Experiments on mice were carried out using mice of wild-type C57BL/6j background, mice with glial conditional deletion of connexin 43 (Cx43) in astrocytes Cx43fl/fl:hGFAP-Cre (Cx43KO, provided by K. Willeke (University of Bonn, Germany) and constitutive knockout mice for Cx30. Experiments were performed in the light phase between 0800 and 1200 h. They were approved by the government of Unterfranken, and performed in accordance with EU guideline 2010/63/EU and with the regulations of the local animal welfare committee (certificate A751901).

### Guide cannula implantations

To study effects of intracerebroventricularly (*icv*) or locally applied synthetic OXT, rats underwent stereotaxic surgery for guide cannula implantation [13] (for details, please see supplementary methods).

### Microinfusions

For examination of acute effects of OXT on astrocytes, rats received a single *icv* infusion of either vehicle (Veh, sterile Ringer solution, pH 7.4, 5 µl) or synthetic OXT (1 nmol/5 µl; [11]) via a 30 G infusion cannula inserted into the implanted guide cannula.

To induce astrocyte-specific knockdown of either *Oxtr* or *Gem* mRNA within the PVN, rats received bilateral intra-PVN microinfusions of respective AAV6-GFAP::shRNA constructs or a control vector expressing a scrambled RNA oligonucleotide via a pulled electrophysiology glass-pipette 21 days prior to behavioral testing (Supplementary Fig. S1a; 280 nl/PVN, 3.28 × 10^13^ GC/ml, stereotaxic coordinates: AP: −1.8 mm bregma, ML: +/−0.4 mm, DV: +8.0 mm; custom designed on www.vectorbuilder.com). The transfected shRNAs were screened in primary rat cortical astrocytes for knockdown efficiency prior to use in vivo (Supplementary Fig. S2).

For assessment of OXT-induced anxiolysis following astrocytic Gem or OXTR knockdown in the PVN, rats received single bilateral OXT infusions into the PVN (0.01 nmol/0.5 µl, i.e. 100-fold lower amount of OXT in a smaller volume than *icv* infusion to prevent tissue damage) 21 d after local virus infusion and 10 min prior to behavioral testing [11]. Correct infusion sites were verified by local ink infusion on 40-µm cryocut, Nissl-stained slices. Subsequently, 4 animals had to be excluded from statistical analysis due to misplaced infusions.

### Behavioral testing

Rats were tested for anxiety-like behavior in the elevated plus-maze (EPM) and light-dark box (LDB), as previously described [11, 13]. To exclude biasing effects of manipulations on locomotive or exploratory behavior, the open field test (OF) was performed. Group sizes of n = 6–11 animals were chosen based on our previous studies on the anxiolytic effect of OXT [11, 13]. The investigator performing the behavioral test including behavioural analyses was blinded to pre-treatment and treatment. For quantification of times spent i) in open and closed arms of the EPM and ii) in the light box of the LDB, and the distance travelled in the OF EthoVision XT tracking software (Noldus Information Technology, Inc.) was used. As indicator of anxiety-related behaviour the percentage of time and the time spent in the open arms of the EPM and the light box of the LDB, respectively, are presented (Fig. 5g, h).

### Acute mouse hippocampal ex vivo slices

Hippocampi from WT and Connexin knockout mice were fixed to a block of agar, on which slices (350 µm) were prepared in a vibratome in oxygenized ACSF. Following bath application of either Veh (ACSF) or OXT (500 nM) for 10 min, slices were fixed in 4% PFA in PBS for 2 h followed by 2 h of blocking in PBS containing 2 g gelatine/l and 1% Triton-X and stained (see *immunohistochemistry)* for morphological analysis, as well as 3D-reconstruction and determination of astrocyte-synapse spatial relationship (see below).

### Cells

#### Primary rat cortical astrocytes

Cells isolated from cortices of newborn rat pups (post-natal day 1–3) were used for all in vitro experiments on astrocytes (adapted from [25]; for details see supplementary methods).

#### H32 neuronal cells

The immortalized fetal rat hypothalamic cell line H32 [26] was cultured at 37 °C and 5% CO2 in DMEM F-12 Ham (Sigma Aldrich; D8437) containing 10% FBS and 1% penicillin/streptomycin. Cells were regularly tested for mycoplasma contamination.

### Transfection of astrocytes by electroporation

In order to study the involvement of Cx43, Sp1 or Gem in astrocytic OXTR signaling, astrocytes were transfected with respective siRNA oligonucleotides (Cx43 (*Gja1*) siRNA, sc-60008, Santa Cruz Biotechnology, Dallas, USA; *Sp1* siRNA, Santa Cruz, sc-61895; *Gem* siRNA, Origene, Rockville, USA, SR507514) or a *Gem* overexpression plasmid (Supplementary Fig. S1b VectorBuilder) by electroporation (Neon™ Transfection System, ThermoFisher; MPK5000).

To screen for shRNA knockdown efficiency in vitro, astrocytes were transfected with a plasmid expressing a shRNA oligonucleotide under control of the long fragment of the *hGFAP* promoter targeted against *Gem* (Supplementary Fig. S1c, VectorBuilder) or *Oxtr* (Supplementary Fig. S1d, VectorBuilder) mRNAs. In case of siRNAs, a scrambled oligonucleotide (scrRNA) served as a control transfection, while a plasmid expressing solely the fluorescent reporter protein EGFP (Supplementary Fig. S1e, VectorBuilder) served as control for plasmid transfections.

### RNA isolation

Cells were trypsinized at ~90% confluency, and RNA was isolated with 1 ml peqGold® TriFast (peqLab, Erlangen, Germany). A subsequent DNA digestion was performed with a DNAse I system (ThermoFisher; EN0521). RNA quantity and quality were determined with a NanoDrop spectrophotometer (ThermoScientific, Waltham, USA).

### Reverse transcriptase PCR, endpoint PCR and quantitative PCR (qPCR)

Reverse transcription was performed with Super Script IV (200U/μl; Life Technologies) system. Primers were designed using the NCBI primer Basic Local Alignment Search Tool (BLAST) software. Primer sequences are listed in Supplementary Table S1. Primer pairs spanned exon-exon junctions to avoid amplification of genomic DNA (for details of endpoint PCR and qPCR-conditions, see supplementary methods).

### Protein extraction

Proteins were either isolated from fresh brain tissue (PVN, amygdala, hippocampus) or cell culture. Pellets/punches were lysed in RIPA buffer (Sigma Aldrich) for 45 min, and debris was removed by centrifugation (13,200 × *g*, 4 °C). Protein concentration was measured with a colorimetric BCA protein assay kit (Pierce ^TM^ BCA Protein Assay Kit, Thermo Scientific) and quantified using an optical density reader (FLUOstar OPTIMA, BMG Labtech, Ortenberg, Germany).

### SDS-PAGE and Western blot

Proteins were separated and quantified as previously described [27, 28]. For details see supplementary methods.

### Immunocytochemistry

Astrocytes (14 d post-enrichment) were seeded in four-chamber glass slides (7 × 10^5^ cells/chamber; Corning; 354104). After 1 h of serum starvation, cells were stimulated with varying concentrations of OXT (1 nM - 1 µM) for the full duration of 10 min or 3 h. Cells were fixed with 4% PFA for 10 min and blocked for 30 min (0.1% TritonX-100, 1% FBS, 10% normal goat serum in PBS). Next, specimen was incubated with primary antibodies (Supplementary Table S2) diluted in PBS containing 0.5% TritonX-100 and 3.3% FBS for 2 h at RT. Appropriate secondary antibodies (Supplementary Table S2) were applied for 2 h at RT in the dark. Slides were covered with ProLong® Gold DAPI (Cell Signaling Technology, Princeton, USA; cs8961).

### Immunohistochemistry

Ten or 20 min after *icv* OXT rats were transcardially perfused with 4% PFA in PBS. Brains were harvested and post-fixed in 4% PFA for 3 h followed by cryo-protection in 30% sucrose for 2 days and snap-freezing in isopentane. Frontal 40-µm cry-sections were prepared, blocked in PBS containing 2% goat serum and 1% TritonX-100 for 1 h, and incubated with primary antibody (Supplementary Table S3) at 4 °C overnight. Appropriate secondary antibodies (Supplementary Table S3) were added for 2 h, and sections were mounted with ProLong® Gold DAPI (Cell Signaling).

### Gap-junctional intercellular communication (GJIC)

To investigate the effects of OXT on astrocytic intercellular coupling, scrape loading dye transfer experiments were performed. 8 × 10^5^ primary astrocytes (14 days in vitro) were seeded in poly-D-lysine coated 35-mm TC dishes (Corning; CLS3294) 2 d prior to the experiments. After 1 h of serum starvation, OXT (1nM–1 µM) or AVP (1nM-1µM) was added to the medium for the full duration of differing timespans (5–180 min). To investigate the underlying signaling cascades, cells were pre-incubated with either 1 µM U0126 (MAP kinase inhibition), 10 µM Gö6983 (PKC inhibition), 1 µM L368,889 (OXTR antagonism), 1 µM carbenoxolone (gap-junction inhibition; [29], Sigma Aldrich; C4790) or Veh (Ringer’s solution) 1 h prior to OXT stimulation. Dishes were rinsed three times with Ca^2+^-free PBS to prevent uncoupling of the cells. Next, 1 ml of warmed (37 °C) lucifer yellow (1 mg/ml in Ca^2+^-free PBS, Sigma Aldrich; L0259) or, in case of EGFP expressing cells, biocytin (1 mg/ml in Ca^2+^-free PBS, Sigma Aldrich; B4261) was added, and three cuts were made through the cell layer with a rounded surgical blade, allowing the fluorescent solution to diffuse within the astrocytic network. After 10 min of incubation, cells were fixed with 4% PFA. For Biocytin experiments, an AlexaFluor594-conjugated streptavidin (ThermoFisher; S32356) was used to visualize Biocytin diffusion. The fluorescence signal was viewed using an epifluorescence microscope (Leica dm5000b), and images of each cut were taken. The fluorescent dye spread area was quantified with ImageJ software (Version 1.52e). Results were regularly cross-validated by an independent investigator blinded to the treatment.

### Bioimaging and image analysis

For all experiments, microscopy and image analysis settings were kept identical within one experiment. In vitro experiments were replicated at least three times.

#### Morphological analysis in vitro, in vivo and ex vivo

To assess effects of OXT and its downstream effectors on astrocytic morphological features, astrocytes were stained for GFAP and DAPI, and images were taken with either Leica SP8 (for in vitro and in vivo) or SP6 (for ex vivo) CLSM. In case of cultured cells, five pictures throughout one culture chamber were acquired per treatment condition and analyzed with ImageJ (Supplementary Fig. S3a). For in vivo and ex vivo analyses, three z-sections per animal and brain region (30 µm, 0.5 µm/z-section, 1024 × 1024) were acquired and analyzed with ImageJ (Supplementary Fig. S3b). Results were regularly cross-validated by an independent investigator blinded to the treatment.

#### 3D-reconstruction of GFP-expressing astrocytes

To analyze OXT-induced changes in volume and surface area of astrocytes, mice received an unilateral intra-hippocampal (CA1 region) infusion of vector plasmid solution containing AAV2/5-GFAP::GFP in PBS (titer 1 × 10^13^ GC/ml,1 µl). After 14 days, acute hippocampal slices (350 µm) were prepared as described. 3D-reconstruction was performed using IMARIS software (Version 9.3, Bitplane AG, Zürich, Switzerland). Two astrocytes per z-section were randomly selected (Supplementary Fig. S4a), and a region of interest (ROI) was created in 3D around these cells (Supplementary Fig. S4b, c). Next, a 3D object was generated within these ROIs (Supplementary Fig. S4d, e), allowing quantification of both cellular surface and volume.

#### Determination of astrocyte-synapse spatial relationship by STED

To assess OXT effects on synaptic spatial relationships, STED nanoscopy was performed on acute ex vivo slice preparations using a custom-built STED microscope (Abberrior/Scientifica) [30]. Synaptic distance to the closest astrocytic element was quantified with a Fiji-Plugin (provided by Philippe Mailly, CIRB imaging facility, College de France, Paris) only including synapses that a) showed no wider distance than 300 nm between pre-and postsynaptic element, and b) contained Homer1/VGlut1 fluorescence maxima in both, deconvolved confocal images and STED images.

#### Colocalization studies

To assess the degree of Cx43 localization at cell/cell-contacts, the tight-junction protein ZO1 was used as a marker for points of intercellular contact [31]. The number of Cx43/ZO1-immunoreactive punctae was determined manually to ensure inclusion of points solely located at cellular contact zones.

#### Intensity measurements and determination of above threshold cells

For immunofluorescence intensity measurements and maxima quantification, images were taken with a Leica SP8 CLSM (63x objective, 16-Bit, 1024 × 1024 pixels) and analyzed with ImageJ. Following background subtraction, a ROI was manually generated around cells of interest and fluorescence intensity was measured. Determination of above threshold cells was accomplished by use of the find maxima function of ImageJ on a background subtracted single image (in vitro experiments) or a sum z-projection (in vivo experiments; 30 µm, 0.5 µm/z-section, 1024 × 1024). Above threshold cells were defined as single cells marked by DAPI staining displaying at least one maximum of the fluorescence of interest.

### Statistical analysis

For statistical analysis, GraphPadPrism (V.8, GraphPad Software, San Diego, USA) was used. Data were first tested for normal distribution by Shapiro–Wilks-test. In case of normal distribution, statistical hypothesis testing was carried out by two-tailed Student’s *t* test (comparison of two treatments/group), one-way (factor: treatment; comparison of more than two treatments) or two-way (factors: pre-treatment and treatment; comparison of pre-treated groups with following treatment) ANOVA, followed by Bonferroni post-hoc analysis, when appropriate. For non-normally distributed data, statistical hypothesis testing was carried out by two-tailed Mann–Whitney U-test (comparison of two treatments/group) or Kruskal–Wallis-test (comparison of more than two treatments) followed by Dunn-Bonferroni post-hoc analysis, when appropriate. All data are presented as mean absolute or relative values +/−SEM or median + min/max, and significance was accepted at *p* < 0.05. *p < 0.05, **p < 0.01, ***p < 0.001 vs. the respective Veh group for i) Student’s *t* test, Bonferroni-post-hoc analysis of one-way ANOVA, Mann–Whitney U-test and Dunn-Bonferroni post-hoc analysis of Kruskal–Wallis test or ii) vs. the respective control group pre-treated with either Veh or scrRNA for Bonferroni post-hoc analysis of two-way ANOVA.

## Results

### Effects of OXT on astrocytic signaling cascades and proteins in vitro and in vivo

**Fig. 1:**
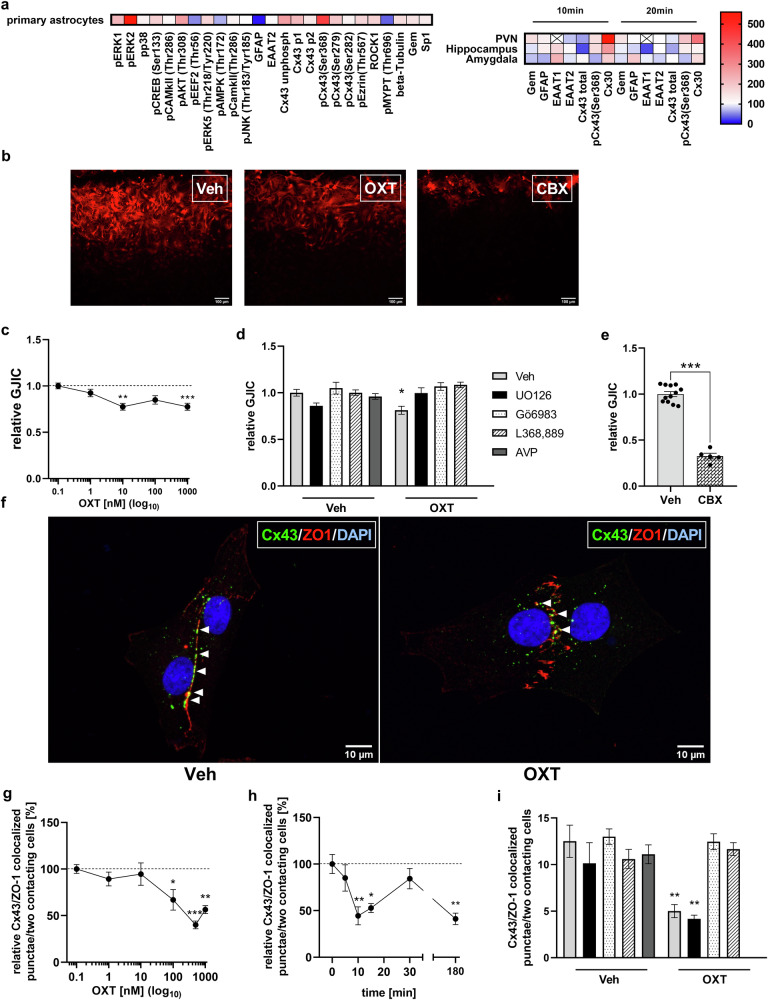
OXT affects astrocytic proteins in vitro and in vivo, and impairs gap-junctional intercellular communication (GJIC) in primary rat cortical astrocytes in a MEK, PKC and OXTR-dependent manner. **a** Heatmap of percentage changes in protein or protein phosphorylation levels in primary astrocytes following 10-min exposure to 500 nM OXT (upper panel), and in brain tissue punches taken from the PVN, hippocampus and amygdala 10 min or 20 min after *icv* administration of OXT (1 nmol/5 µl) (lower panel). Downregulations are colored in blue, while upregulations are colored in red. **b** Representative images of streptavidin staining visualizing the distance of biocytin diffusion and, thus, reflecting GJIC within the astrocytic network. Exposure of primary astrocytes to either OXT (500 nM) or the gap-junction blocker carbenoxolone (CBX, 1 µM) impaired relative GJIC compared to vehicle (Veh) treatment. Scale bar = 100 µm. **c** Dose-dependent effects of OXT (applied for 10 min) on relative GJIC in primary astrocytes (treatment F_4_,_73_ = 6.847, *p* < 0.001 versus Veh; **p = 0.001 for 10 nM; ***p = 0.0004 for 1000 nM). **d** Relative GJIC of astrocytes pre-treated for 1 h with either Veh, the MAP kinase inhibitor U0126 (1 µM), the PKC inhibitor Gö6983 (10 µM) or the OXTR antagonist L368,889 (1 µM) prior to OXT exposure (500 nM, 15 min) or AVP exposure (1 µM, 15 min) (interaction: F_3_,_62_ = 5.574, *p* = 0.002; *p = 0.01 Veh/OXT vs. Veh/Veh treatment). **e** Relative GJIC in astrocytic cultures after treatment with CBX (t_15_ = 14.75, ***p < 0.0001 versus Veh). **f** Representative immunocytochemical images of astrocytes stained for Cx43 (green), the tight-junction protein ZO1 (red) and DAPI (blue) showing lower levels of Cx43/ZO1 colocalization in OXT- compared with Veh-treated cells. White arrows indicate points of Cx43/ZO1 colocalization. **g** Dose-dependent effects of OXT (15 min) on Cx43 localization at cell-cell contacts (treatment F_5,36_ = 8.810, *p* < 0.001 versus Veh; *100 nM: *p* = 0.050, ***500 nM: *p* = 0.0002, **1 µM: *p* = 0.003). **h** Effect of differing exposure times to OXT (500 nM) on Cx43 localization at cell-cell contacts (treatment F_5,36_ = 6.077, *p* < 0.001 vs. basal (t = 0); **p = 0.005 for 10 min; *p = 0.024 for 15 min; **p = 0.003 for 180 min). **i** Impact of OXT (500 nM, 15 min) or AVP (1 µM, 15 min) on Cx43/Zo1 colocalization following pre-treatment with either Veh, U0126, Gö6983 or L368,889 (see legend in D) (interaction: F_3,46_ = 5.267, *p* = 0.003; **p = 0.008 for Veh/OXT vs. Veh/Veh; ** *p* = 0.001 U0126/OXT vs Veh/Veh). Data represent mean absolute or relative values +/−SEM.

We focused on brain regions, such as the hypothalamic PVN, hippocampus and amygdala, and signaling cascades, which have been previously linked to neuronal OXTR activation [1, 11, 13, 32], as well as on targets preferentially or exclusively expressed in astrocytes. Our experiments revealed acute effects of OXT on components of various signaling pathways including MAPK signaling (pCREB, pERK1/2 and pERK5), cytoskeleton-associated pathways (Gem, Sp1, beta-tubulin and ROCK1) and pathways related to intercellular connectivity (Cx30 and Cx43). In detail, in primary astrocytes (see Fig. 1a), exposure to OXT for 10 min increased the cytoskeleton-related proteins pEzrin/Thr567, beta-Tubulin, ROCK1 and the endogenous ROCK-inhibitor Gem, while pMYPT/Thr696 and GFAP were reduced. The astrocytic glutamate transporter EAAT2 was unaffected. Furthermore, OXT exposure increased the phosphorylation level of the signaling proteins pCreb/Ser133, pAkt/Thr308, pERK1/2, and pERK5/Thr218/Tyr220, while decreasing peEF/Thr56 phosphorylation. No changes were observed for pp38, pAMPK/Thr172, pJNK/Thr183/Thr185 and pcamKII/Thr286. Furthermore, OXT-exposure elevated phosphorylation levels of gap-junction proteins (pCx43/Ser368, pCx43/Ser279, pCx43/P1, and pCx43/P2).

The alterations in protein and protein phosphorylation levels observed in vivo were time and brain region-dependent (see Fig. 1a). Particularly, in line with the effects of OXT on cortical astrocytic cytoskeleton- and gap junction-related proteins in vitro, *icv* OXT increased levels of Cx30, pCx43/Ser368 and Gem within the PVN after 10 min, while downregulating EAAT2 and unphosphorylated Cx43. Elevation of Cx30, pCx43/Ser368 and Gem remained observable after 20 min, whereas EAAT2 and unphosphorylated Cx43 levels recovered to control levels. Within the hippocampus, *icv* OXT resulted in elevated quantities of Cx30 and pCx43/Ser368 after 10 min, decreased quantities of Cx43 after 10 min to 20 min, and in downregulation of the astrocytic glutamate transporter EAAT1 at 20 min. Within the amygdala, *icv* OXT increased Cx30 and EAAT1, while decreasing Gem levels after 10 min. After 20 min, upregulated GFAP and decreased quantities of EAAT2 were found. For a summary of all examined target proteins and detailed statistics see Fig. 1a and Supplementary Table S4.

### Effects of OXT on the expression of selected astrocytic genes

Based on the OXT-induced changes in astrocytic protein levels, we analysed the dynamics of corresponding mRNA expression in primary cortical astrocytes (Supplementary Fig. S5 and Supplementary Table S5). Briefly, after 10 min, OXT tended to decrease mRNA levels of Cx30 (*Gjb6*) and Cx26 (*Gjb2)* (Supplementary Fig. S5a), whereas Cx43 (*Gja1*) mRNA was not affected. After 30 min, the trend of decreased *Gjb2* expression observed at 10 min became significant. Simultaneously, an increased *Gja1* as well as *Gem* expression was detected, while EAAT2 (*Slc1a2)* expression remained unchanged (Supplementary Fig. S5b). Contrary to a shorter exposure, 180 min of OXT stimulation caused a decrease in *Gja1* expression (Supplementary Fig. S5c), while *Gjb2* mRNA recovered to control levels. Last, *Gjb6* expression was still lowered after 180 min.

### Effects of OXT on distribution of astrocytic gap-junction proteins and intercellular connectivity

Based on the OXT-induced downregulation of astrocytic gap-junction subunits both at mRNA and protein level, we next tested, whether OXT affects gap-junctional intercellular communication (GJIC) using dye-spread assays (Fig. 1b; [33]). The broad-range gap-junction inhibitor carbenoxolone served as a positive control (Fig. 1b, e; [29]). In an initial dose-response experiment, OXT acutely impaired GJIC at doses of 10 nM and 1 µM (Fig. 1c). OXT-treated cells showed impaired GJIC by around 20%, while this effect was prevented by OXTR/MEK/PKC inhibitors/antagonists (Fig. 1d). The related nonapeptide arginine vasopressin (AVP) had no effect on GJIC (100 nM; Fig. 1d).

To visualize the impact of OXT on astrocytic interconnectivity on a single cell level, we co-stained the most abundant astrocytic gap-junction protein Cx43 with the tight-junction protein ZO1 as marker for cell-cell contacts (Fig. 1f). OXT at 100 nM, 500 nM and 1 µM reduced Cx43 localization at cell-cell contacts by 35–60% (Fig. 1g; Supplementary Fig. S5d) beginning at 10 min post-application (Fig. 1h). A longer exposure time (180 min) similarly reduced Cx43 localization.

To investigate the underlying signaling cascades of reduced Cx43 localization at cell-cell contacts by OXT, cells were pre-treated as described for GJIC experiments prior to OXT stimulation (500 nM; 15 min). OXT-treated cells displayed less Cx43 localization at cell-cell contacts, and this effect was PKC and OXTR-dependent, but not MEK- dependent (Fig. 1i). AVP had no effect (100 nM; Fig. 1i).

### OXT-induced changes in astrocytic cytoskeletal dynamics in vitro and in vivo and astrocyte-neuron spatial relationships

**Fig. 2:**
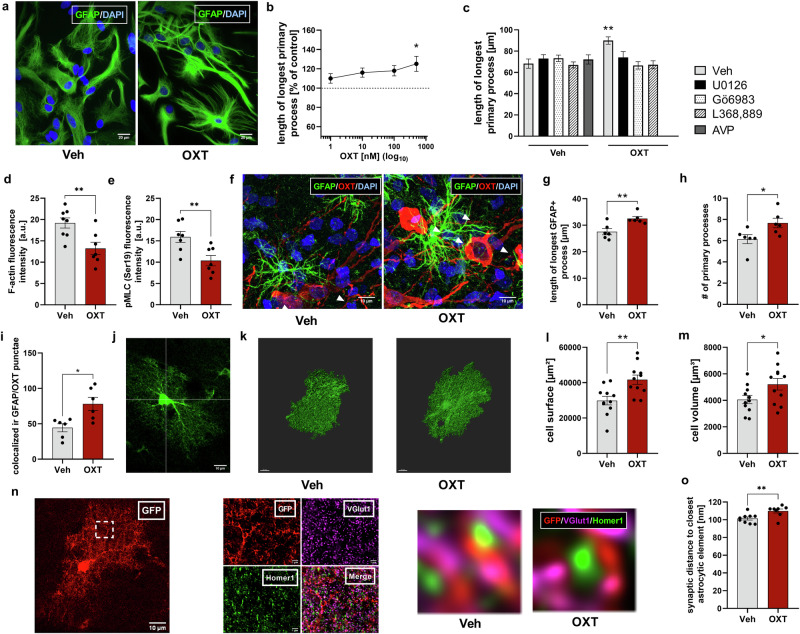
OXT alters the astrocytic morphological phenotype in a PKC, MEK and OXTR-dependent manner and astrocyte-neuron spatial relationships in vitro (in primary cortical rat astrocytes) and in vivo. **a** Representative images of primary astrocytes stained for GFAP (green) and DAPI (blue) showing OXT-induced morphological alterations. Scale bar = 20 µm. **b** Dose-dependent effect of 10-min exposure to OXT on the length of the longest primary process in vitro (F_4,539_ = 2.760, *p* = 0.027; *p = 0.016 for 500 nM OXT vs. Veh). **c** Effect of 10-min OXT or AVP exposure on process length of primary astrocytes following pre-treatment with either Veh, U0126, Gö6983 or L368,889 (for details see legend to Fig.1; interaction: F_3,646_ = 4.480, *p* = 0.004; **p = 0.008 Veh/Veh vs. Veh/OXT. **d**, **e** in vitro effects of 180-min OXT exposure (**d**) on astrocytic F-actin levels reflected by phalloidin immunofluorescence (t_13_ = 3.225, **p = 0.007 vs. Veh), and (**e)** on phospho(Ser19)-myosin-light-chain-kinase (pMLC/Ser19) immunofluorescence (t_12_ = 3.136, **p = 0.009 vs. Veh). **f** Representative images of PVN astrocytes (GFAP; green) co-stained with neurophysin (OXT; red) and DAPI (blue) in rats 10 min after *icv* Veh or OXT (1 nmol/5 µl). White arrows mark points of GFAP/neurophysin (OXT) colocalization indicating astrocyte-neuron contacts. Scale bar = 10 µm. **g**–**i** Effects of *icv* infusion of OXT on primary process length after 10 min (**g**; t_10_ = 3.48, **p = 0.006), primary process number (H; t_10_ = 2.47, *p = 0.033) and GFAP/OXT-colocalization (**I**; t_10_ = 3.09, * *p* = 0.011) within the rat PVN. **j** Representative confocal microscopy image of a GFP-expressing mouse hippocampal astrocyte. Scale bar = 10 µm. **k** Representative 3D reconstructions of astrocytes created from Veh or OXT (500 nM, 10 min) -treated acute mouse hippocampal slices. Scale bar = 10 µm. **l** Assessment of cellular surface area from 3D reconstructed astrocytes (t_20_ = 3.302, **p = 0.004). **m** Assessment of cellular volume from 3D-reconstructed astrocytes (t_20_ = 2.155, *p = 0.046). **n** Representative confocal microscopy image of a GFP-expressing mouse hippocampal astrocyte. Scale bar = 10 µm. White dotted box indicates inlay for middle panel. Inlay includes deconvolved confocal image of astrocytic element (GFP; red), as well as deconvolved STED-images of pre-synaptic (VGlut1; Magenta) and post-synaptic (Homer1; green) markers. Scale bar for inlays = 1 µm. Right panel displays representative astrocyte processes in their spatial relationship to pre- and post-synaptic elements from slices treated with either Veh or OXT. **o** Average synapse/astrocyte distance per analyzed inlay (t_15_ = 3.003, **p = 0.009). Data represent mean absolute or relative values +/−SEM.

The modulation of neuronal communication by astrocytes highly depends on the spatial relationship of astrocytes and neuronal synapses, critically set by the astrocytic cytoskeleton. Since we revealed changes of several proteins associated with cytoskeletal dynamics, we examined OXT effects on the cytoskeleton of astrocytes in vitro and in vivo. In an initial dose-response experiment (Fig. 2a, b), 500 nM OXT caused a rapid elongation of GFAP+ astrocytic processes after 10 min in cultured cells. To investigate the underlying signaling mechanisms, cells were pre-treated as described above prior to 10 min of OXT exposure. In the subsequent analysis of primary process length and number, OXT-treated cells showed an increased length of primary processes by around 25%, an effect blocked by pre-administration of either the OXTR antagonist, or the MEK or PKC inhibitors (Fig. 2c). Differences in process number (Supplementary Fig. S6a) were found with OXT causing a ~30% increase in primary process number that was MEK, PKC, as well as OXTR-dependent. Similar to gap-junction experiments, AVP was without effect (100 nM; Fig. 2c; Supplementary Fig. S6c). OXT stimulation for a prolonged period (180 min) produced comparable results (Supplementary Fig. S6b–d).

Since cellular process formation and elongation are indicators of a dampened activity of the RhoA/ROCK pathway [34–36], and considering that our protein analyses revealed an OXT-induced increase in the endogenous RhoA/ROCK inhibitor Gem as well as a decrease of the ROCK phosphorylation-target pMYPT/Thr696, F-actin (Phalloidin) and phospho(Ser19)-myosin-light-chain-kinase, fluorescent intensity measurements were used as additional indirect markers of RhoA/ROCK activity (Supplementary Figs. S6e; [37]). Indeed, prolonged OXT stimulation (500 nM, 180 min) induced a decrease in F-actin stress fibres (Supplementary Figs. 2d, S6e) and pMLC/Ser19 levels (Supplementary Figs. 2e, S6e), both indicative of a dampened RhoA/ROCK activity. Taken together, OXT rapidly modulates the cytoskeleton of astrocytes in vitro.

To validate these effects in vivo, PVN and hippocampal (CA1 region) astrocytes were examined 10 min after *icv* OXT for changes in cellular morphology and resulting neuron-astrocyte spatial relationships. Corroborating in vitro experiments, OXT caused GFAP+ astrocytic process elongation (Fig. 2f, g) and ramification (Fig. 2f, h) within the PVN, leading to an increased astrocytic coverage of OXT neurons (Fig. 2f, i). The total amount of PVN GFAP+ cells remained unchanged (Supplementary Fig. S6f).

As GFAP is a cytoskeleton protein not expressed throughout the entire astrocytic cell, we next used a viral vector-based strategy to express GFP under the promoter of the *hGFAP* gene (Fig. 2j). 3D-reconstruction (Fig. 2k) revealed an increase in astrocyte surface (Fig. 2l) and volume (Fig. 2m) after acute (10 min) OXT exposure. Co-staining with pre-/ post-synaptic markers together with STED nanoscopy (Fig. 2n; Supplementary Fig. S6g) revealed an OXT-induced increase in the spatial relationship between astrocytes and excitatory synapses after 10 min of bath application in acute hippocampal slices (Fig. 2o), suggesting an effect of OXT-induced morphological remodeling on neuronal communication.

### The involvement of the small GTPase Gem in astrocytic OXT effects

**Fig. 3:**
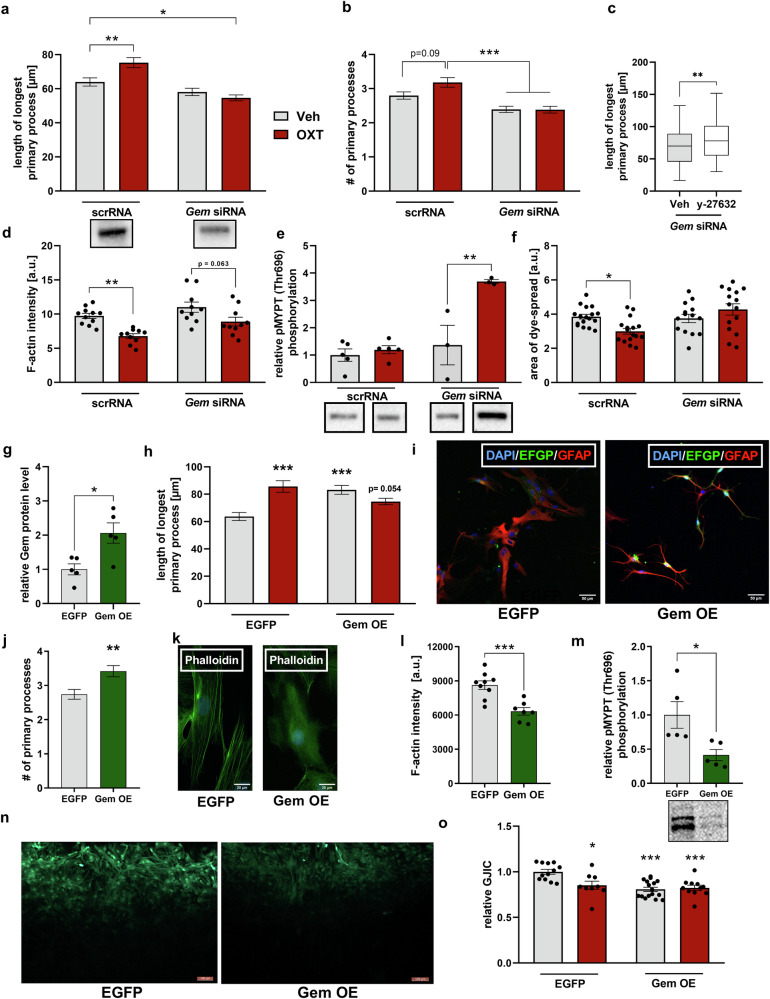
Gem is required and sufficient for the effects of OXT on astrocytes in vitro. **a**, **b** Gem siRNA knockdown prevents OXT-induced prolongation of primary processes (**a**; interaction: F_1,405_ = 10.58, *p* < 0.001; **p = 0.008 scRNA/Veh vs. scRNA/OXT; *p = 0.016 siRNA/OXT vs. scrRNA/Veh) and increase in primary process number (**b**; interaction: F_1,491_ = 3.204, *p* = 0.074; *p* = 0.09 for scRNA/Veh vs. scRNA/OXT; ***p < 0.001 siRNA-pretreated vs. scRNA-pretreated/OXT astrocytes). Cells were transfected with either *Gem* siRNA or a control oligonucleotide (scrRNA) 48 h prior to OXT (500 nM, 10 min). Representative Western blot bands of successful knockdown (scRNA/Veh vs. siRNA/Veh) are shown below. **c** Length of longest primary process after exposure to the ROCK-inhibitor y-27632 (1 µM, 30 min) in primary astrocytes transfected with *Gem* siRNA (*U* = 5936, **p = 0.009). **d** Phalloidin/F-actin immunofluorescence after Gem knockdown and subsequent OXT exposure (500 nM, 10 min; interaction: F_1,37_ = 0.588, *p* = 0.448; **p = 0.003 scrRNA/Veh vs. scrRNA/OXT, *p* = 0.063 siRNA/Veh vs. siRNA/OXT). **e** Relative phosphorylation of MYPT at Thr696 following Gem knockdown and subsequent OXT stimulation (interaction: F_1,12_ = 15.42, *p* = 0.006; **p = 0.003 siRNA/Veh vs. siRNA/OXT). Representative Western blot bands are shown below. **f** Absolute area of lucifer yellow diffusion (dye spread experiments) after Gem knockdown and subsequent OXT exposure (interaction: F_1,59_ = 9.424, *p* = 0.003; *p = 0.041 scrRNA/Veh vs. scrRNA/OXT). **g** Gem protein levels in primary astrocytes transfected with either EGFP control plasmid or Gem overexpression (OE) plasmid indicating successful Gem OE after 72 h (t_8_ = 3.137, *p = 0.014). **h**–**j** Effects of Gem OE on the cytoskeleton of astrocytes reflected by increased process length (**h**; interaction: F_1,354_ = 23.64, *p* < 0.001; ***p < 0.001 EGFP/Veh vs. Gem OE/Veh and vs. EGFP/OXT) and process number (**J;** *U* = 1881, **p = 0.002). Representative images taken from EGFP-expressing control or Gem OE cells stained for DAPI (blue) and GFAP (red) (**I**; scale bar = 50 µm). **k** Representative images of phalloidin staining in EGFP vs. Gem OE -transfected cells. Scale bar = 20 µm. **l** Phalloidin/F-actin immunofluorescence intensity in EGFP vs. Gem OE -transfected cells (t_14_ = 4.467, ***p < 0.001). **m** pMYPT/Thr696 phosphorylation levels reflecting ROCK activity in EGFP vs. Gem OE -expressing cells (t_8_ = 2.792, *p = 0.026). **n** Representative images of scrape-loading dye transfer experiments in EGFP vs. Gem OE -transfected cells. Scale bar = 100 µm; biocytin-AlexaFluor594 streptavidin staining pseudocolored in green. **o** Degree of GJIC in EGFP vs. Gem OE -transfected cells followed by stimulation with OXT (500 nM, 10 min) (interaction: F_1,44_ = 7.450, *p* = 0.009; ***p < 0.001 EGFP/Veh vs. Gem OE/Veh and vs. Gem OE/OXT; *p = 0.018 EGFP/Veh vs. EGFP/OXT). Data represent mean relative or absolute values +/−SEM for normally distributed data and median +/−min/max values for non-normally distributed data.

Considering the OXT-induced upregulation of the endogenous ROCK-inhibitor Gem and simultaneously observed dampened activity of the RhoA/ROCK pathway, we next tested the hypothesis that Gem is central in conveying the effects of OXT on astrocytes. Therefore, we knocked down Gem in vitro (Supplementary Fig. S7a), which prevented OXT-induced process elongation. siRNA/OXT-treated cells even displayed shortened processes compared to scrRNA/Veh-treated cells (Fig. 3a). OXT did induced ramification in scrRNA control cells by statistical trend, as *Gem* siRNA/OXT cells displayed significantly fewer primary processes than scrRNA/OXT-treated cells (Fig. 3b). We could further exclude the possibility that the cytoskeleton of *Gem* siRNA cells is uncapable to respond to stimuli: treatment of *Gem* siRNA cells with the ROCK-inhibitor y-27632 (1 µM for 30 min; [38]) as positive control resulted in a process elongation (Fig. 3c). Furthermore, the observed OXT-induced breakdown of F-actin stress-fibres might also partially be Gem-dependent, as knockdown of Gem blunted this effect (Fig. 3d). Notably, OXT induced ROCK-activity solely in Gem knockdown astrocytes, as assessed by pMYPT/Thr696 phosphorylation levels (Fig. 3e), which might explain the observed retraction of processes in *Gem* siRNA/OXT cells (Fig. 3a). Similar to OXT effects on the cytoskeleton, we found OXT-induced effects on astrocytic gap-junctions to be Gem-dependent, as Gem knockdown prevented OXT-induced (i) impairment of GJIC (Fig. 3f), and (ii) reduction of Cx43 localization at cell-cell contacts (Supplementary Fig. S7b).

In a gain-of-function approach, we next tested whether overexpression (OE) of Gem (Fig. 3g, Supplementary Fig. S7c–e) mimics the effect of OXT on the astrocytic cytoskeleton. Indeed, Gem OE caused significant process elongation and ramification (Fig. 3h–j) to a similar extent as OXT, and OXT had no further add-on effect in Gem OE cells compared to control EGFP expressing cells. Again, similar to OXT, Gem OE elicited significant stress fibre breakdown (Fig. 3k, l), and a strong reduction in ROCK-activity as assessed by quantification of pMYPT/Thr696 phosphorylation levels (Fig. 3m).

In GJIC experiments, Gem OE impaired astrocyte network connectivity with no add-on effect of OXT (Fig. 3n, o). On a molecular level, Gem OE altered Cx43 phosphorylation states (Supplementary Fig. S7f, g) and decreased Cx43 (*Gja1*) mRNA (Supplementary Fig. S7h) analogously to OXT stimulations. Taken together, Gem is required and sufficient for the effects of OXT on astrocytic morphology and intercellular connectivity.

### Involvement of astrocytic connexin 43 in OXT-induced cytoskeletal remodeling

**Fig. 4:**
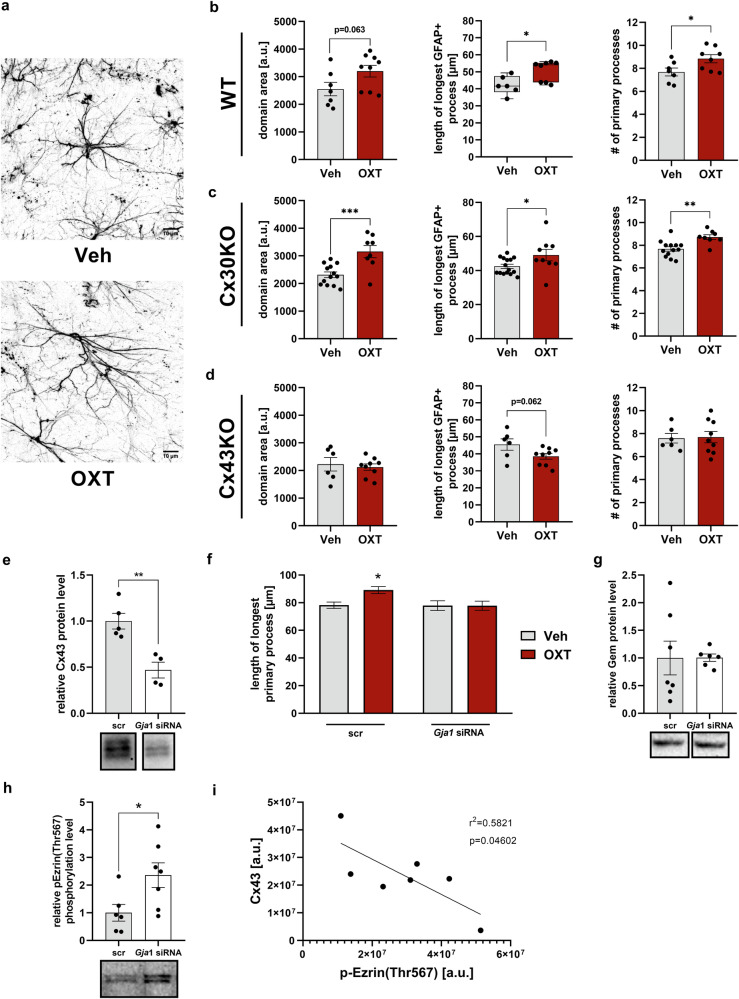
Cx43, but not Cx30, is involved in OXT-induced alterations of the cytoskeleton of astrocytes. **a** Representative images taken from mouse acute hippocampal slices treated with either 500 nM OXT or Veh for 10 min. Scale bar = 10 µm. **b**–**d** Domain area, process length and process number of astrocytes in acute hippocampal slice preparations from either wildtype (WT; **b**), Cx30 knockout (**c**) or Cx43 knockout (**d**) mice treated with OXT or Veh (treatment: domain area: t_14_ = 2.016, *p* = 0.063 WT; t_19_ = 3.900, ***p = 0.001 Cx30KO; process length: *U* = 6, *p = 0.02 WT; t_22_ = 2.183, *p = 0.04 Cx30KO; process number: t_13_ = 2.321, *p = 0.037 WT; t_19_ = 3.456, **p = 0.003 Cx30KO). **e** Validation of successful Cx43 knockdown in rat primary astrocytes by means of immunoblotting (t_7_ = 4.319, **p = 0.004). Representative Western blot bands are shown below. **f** Quantification of longest primary process in cells transfected with either *Gja1* siRNA or a control oligonucleotide (scrRNA) and subsequent administration of OXT (interaction: F_1,680_ = 3.617, *p* = 0.058; **p = 0.01 scrRNA/Veh vs. scrRNA/OXT). **g** No effect of Cx43 knockdown on Gem protein quantities. Representative Western blot bands are shown below. **h** Effect of Cx43 knockdown on phosphorylation of Ezrin/Thr576 reflecting an increase in active Ezrin (t_11_ = 2.438, *p = 0.033). Representative Western blot bands are shown below. **i** Correlation of Cx43 level in *Gja1* siRNA transfected cells with Ezrin phosphorylation (*p* = 0.046, r^2^ = 0.582). Data represent mean relative or absolute values +/−SEM.

To further assess the link between OXT-induced regulation of astrocytic gap-junction proteins and altered cytoskeletal dynamics, acute hippocampal slices prepared from Cx30KO or Cx43KO mice, as well as WT mice (C57BL/6) were treated with 500 nM OXT for 10 min. Similar to experiments in rats, OXT increased process length, process number and domain area in slices from WT and Cx30KO mice (Fig. 4a–c), but not from Cx43KO mice (Fig. 4d). This suggests a selective involvement of Cx43 in OXT-induced cytoskeletal dynamics of astrocytes.

Consistent with these data, Cx43 knockdown in vitro prevented OXT-induced elongation of astroglial processes (Fig. 4e, f). Transfection with Cx43 siRNA did not affect the total amount of Gem (Fig. 4g), but increased the phosphorylated (i.e., active) levels of the Gem effector Ezrin (Fig. 4h), indicating that Ezrin (and likely Gem) is negatively regulated by Cx43 levels (Fig. 4i).

### Regulation of Gem by OXT at the genomic level

To reveal the transcription factor mediating the upregulation of Gem by OXT, we used a publically available database (AliBaba 2.1; http://gene-regulation.com/pub/programs/ alibaba2/index.html) to predict transcription factor binding sites within the promoter region of *Gem*. Here, Sp1 posed the highest number of binding sites (Supplementary Table S6; 12 predicted sites vs. 1-2 for other transcription factors).

**Fig. 5:**
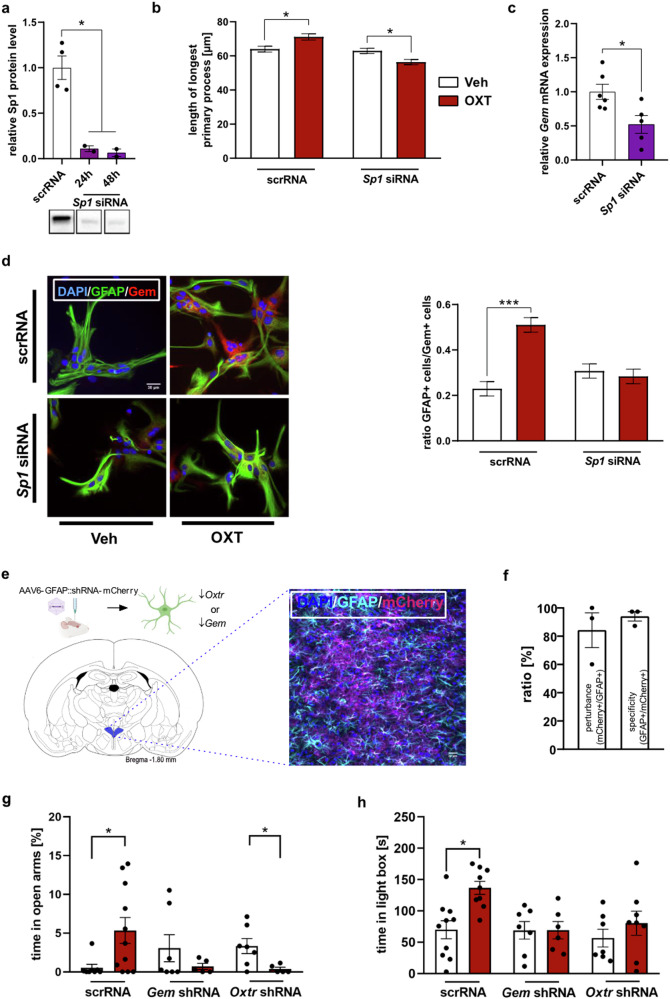
The transcription factor Sp1 conveys OXT-induced Gem expression, and astrocytic OXTR signaling within the PVN is required for the local anxiolytic effect of OXT. **a** Successful knockdown of Sp1 by transfection of primary astrocytes with *Sp1* siRNA assessed by immunoblotting (t_4_ = 4.548, *p = 0.01 at 24 h, and t_4_ = 4.548, *p = 0.009 at 48 h siRNA vs. scrRNA). Representative bands are shown below. **b** Effects of Sp1 knockdown on OXT-induced prolongation of the longest primary process. Primary astrocytes were transfected with either *Sp1* siRNA or a control oligonucleotide (scrRNA) 48 h hrs prior to OXT exposure (500 nM, 10 min). (Interaction: F_1,1106_ = 16.51, *p* < 0.001; *p = 0.012 scrRNA/Veh vs. scrRNA/OXT; *p = 0.031 for siRNA/Veh vs. siRNA/OXT). **c** Effects of Sp1 knockdown on *Gem* mRNA expression (t_9_ = 2.818, *p = 0.02 vs. scrRNA). **d** Representative images from *Sp1* siRNA or scrRNA-transfected astrocytes subsequently exposed to OXT (500 nM, 10 min) and stained for GFAP (green), DAPI (blue) and Gem (red) showing Sp1-dependency of OXT-induced Gem expression. Scale bar = 10 µm. Quantification of above threshold (Gem+) cells was defined as single cells marked by DAPI/GFAP staining displaying at least one maximum of above threshold Gem fluorescence (interaction: F_1,832_ = 22.32, *p* < 0.001; ***p < 0.001 scrRNA/Veh vs. scrRNA/OXT; scale bar = 30 µm). **e** Targeting of astrocytes within the rat PVN with AAV6-GFAP::shRNA vectors to induce either *Oxtr* (*Oxtr* shRNA) or *Gem* (*Gem* shRNA) knockdown in a cell type-specific manner. Illustration adapted from Paxinos and Watson (2006) to show anatomical structures of the rat PVN. Representative image taken from the PVN of rats that received bilateral microinfusions of either *Oxtr*- or *Gem-* knockdown vector (280 nl per side) 21 d before. Successful transfection is indicated by the expression of the fluorescent reporter mCherry, while cell-type specificity is indicated by double positive immunostaining for mCherry and GFAP (scale bar = 20 µm). **f** Quantification of perturbance and specificity of AAV6-GFAP::shRNA constructs (n = 3). **g** Percentage of time spent in open arms of the EPM of rats pre-treated with either *Oxtr* shRNA or *Gem* shRNA for astrocyte-specific knockdown of OXTR or Gem, or with scrRNA within the left and right PVN, and locally infused with either Veh or OXT (0.01 nmol/0.5 µl) 10 min prior testing (genotype F_2_,_37_ = 0.443, *p* = 0.639; treatment F_1_,_37_ = 0.021, *p* = 0.885, *p = 0.045 scr/Veh vs. scr/OXT, *p = 0.030 shRNA/Veh vs. shRNA/OXT; n = 6–11/group). **h** Time spent in the light box of the LDB of rats treated as described above (g), (genotype F_2_,_41_ = 3,965, *p* = 0.027; treatment F_1_,_41_ = 6.165, *p* = 0.017, *p = 0.016 scr/Veh vs. scr/OXT; n = 6–11/group). Data represent mean relative or absolute values +/−SEM.

Based on above in silico analysis and the finding that OXT increased Sp1 protein levels (Fig. 1a), we hypothesized that Sp1 controls Gem expression and conveys OXT-induced Gem-dependent alterations to the cytoskeleton of astrocytes. In-vitro knockdown of Sp1 (Fig. 5a, Supplementary Fig. S8a) prevented OXT-induced process elongation (Fig. 5b). Similar to the OXT-induced retraction of processes following Gem knockdown (Fig. 3a), OXT decreased process length in the Sp1 knockdown group. Importantly, knockdown of Sp1 led to a decrease of *Gem* mRNA expression (Fig. 5c) and prevented OXT-induced upregulation of Gem (Fig. 5d, Supplementary Fig. S8b). However, Sp1 knockdown did not affect *Oxtr* mRNA levels (Supplementary Fig. S8c), excluding the possibility of nonresponsive OXTR-coupled pathways caused by secondary effects on Oxtr expression.

### Involvement of astrocytic OXTR signaling in the acute anxiolytic properties of OXT

Among the many behavioural effects of OXT is its established anxiolytic action within the PVN after local infusion of synthetic OXT [11, 39, 40]. To study the behavioral relevance of astrocytic OXTR signaling and its downstream effector Gem within the PVN, we established AAV6-GFAP::shRNA-mCherry vectors providing an astrocyte-specific knockdown of OXTR or Gem (Supplementary Figs. S1a,  S2). Transfection specificity (ratio mCherry+/GFAP+ cells; 94.0%) and perturbance (ratio GFAP+/mCherry+ cells; 84.3%) was analyzed three weeks post-infection within the PVN (Fig. 5e–f). For assessment of OXT-induced anxiolysis following astrocytic Gem or OXTR knockdown, rats received bilateral OXT infusions 21 d after virus infusions (Supplementary Fig. S8d) and 10 min prior to assessment of anxiety-related behavior in the EPM and LDB.

In both tests for anxiety-related behavior, we confirmed the acute anxiolytic effect of OXT within the PVN shown before [28], as scrRNA/OXT rats showed significantly increased percentage of time spent in the open arms (EPM; Fig. 5g) and increased time spent in the light area (LDB; Fig. 5h) and entered these areas more often (data not shown) than scrRNA/Veh controls. In contrast, these effects were absent in both shRNA/Oxtr- or shRNA/Gem knockdown rats. No group differences in locomotion (EPM, OF) were found (Supplementary Fig. S8f, g). These results indicate that astrocytic OXTR signaling within the PVN is required for the local anxiolytic effect of OXT.

### Differential regulation of the Sp1-Gem-ROCK axis in neuronal cells

**Fig. 6:**
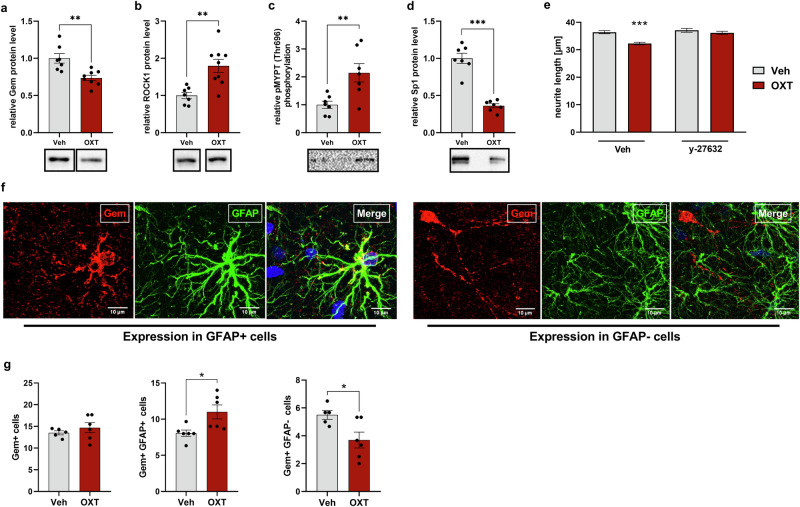
The regulation of Gem by OXT is polarized depending on cell type. Effect of OXT (100 nM, 180 min) on protein levels of Gem (**a**; t_13_ = 3.700, **p = 0.003), ROCK1 (**b**; t_14_ = 3.744, **p = 0.002), pMYPT/Thr696 (**c**; t_12_ = 3.244, **p = 0.007) and Sp1 (**d**; t_12_ = 8.751, ***p < 0.001) in H32 neurons assessed by immunoblotting. Representative bands are shown below. **e** Effects of OXT on neurite length of H32 cells pre-treated with either Veh or the ROCK-inhibitor y-27632 (interaction: F_1,1884_ = 7.396, *p* = 0.007, ***p < 0.001 Veh/Veh vs. Veh/OXT). **f**, **g** Representative images of hippocampal astrocytes (GFAP; green) co-stained with Gem (red) and DAPI (blue) in rats that received either Veh or OXT *icv*. Quantification of above threshold (Gem+) cells was defined as single cells marked by DAPI/GFAP (Gem+/GFAP+; t_10_ = 2.771, *p = 0.020) or DAPI/absence of GFAP (Gem+/GFAP-; t_10_ = 2.606, *p = 0.029) staining displaying at least one maximum of above threshold Gem fluorescence. Data represent mean relative or absolute values +/−SEM.

Since OXT exerts contrary effects on the cytoskeleton of neuronal cells [41], i.e., a retraction of neurites, we examined whether OXT differentially regulates the Sp1-Gem-ROCK signaling axis in H32 neuronal cells. Indeed, in contrast to astrocytes, stimulation of H32 neurons with OXT (100 nM, 180 min) revealed an OXT-induced increase in ROCK1 protein level and ROCK activity and a decrease in Gem and Sp1 levels (Fig. 6a–d; Supplementary Fig. S8g for Gem protein levels after 10 min of OXT exposure). OXT-induced retraction of neurites was prevented in cells pre-treated with the ROCK-inhibitor y-27632 (Fig. 6e).

To support the idea of this cell type-specific effect, the cellular distribution of Gem was analyzed within the hippocampus of *icv* OXT-treated rats (Fig. 6f). In line with protein analyses (Fig. 1a, Supplementary Table S4), OXT did not alter the total amount of Gem (Fig. 6g). However, an analysis of the distribution in astrocytes (GFAP+) vs. non-astrocytic cells (GFAP−) revealed an OXT-induced increase of Gem-positive (Gem+) astrocytes and a simultaneous decrease of Gem-expressing non-astrocytic cells, supporting the idea of a cell type-dependent polarized regulation of Gem by OXT and consequent differing outcomes in cytoskeletal responses.

## Discussion

In this study, we describe the effects of OXT on astrocytic gene expression, intracellular signaling as well as astrocyte-specific proteins both in vitro and in vivo. We further show that OXT induces rapid alterations in the cytoskeletal plasticity of astrocytes, gap-junction coupling and astrocyte-neuron spatial relationships. Specifically, we identify the previously undescribed Sp1-Gem signaling cascade as being central to these cell type-specific effects of OXT. Targeted downregulation of astrocytic OXTR signaling in the rat PVN finally provides evidence that OXT requires direct binding to astrocytes to exert its local anxiolytic effect. Thus, our study establishes astrocytes as a critical component involved in the behavioural effects of the brain OXT system.

Our initial analysis of OXTR-coupled pathways demonstrates the high responsiveness of astrocytes to OXT and points towards Ca^2+^ and EGFR as important nodes of astrocytic OXTR signaling. This is in line with previous studies, in which OXT elicited rapid dose-dependent Ca^2+^ release from intracellular stores in cultured hypothalamic astrocytes [8] and an increase in nuclear pERK1/2 in astrocytes of acute SON slice preparations [42], respectively. These identified downstream activation patterns (Fig. 1a), in general, compare well to OXT effects in neuronal and myometrial cells [11, 13, 43, 44], for review see [1]. However, we also report on astrocyte-specific responses to OXT, such as the lack of CamKII activation [10] and a decrease in the activity of ROCK ([45]).

An important finding is that OXT rapidly impairs astrocytic intercellular coupling via specific signaling proteins, such as MAPKs and ERKs (Fig. 1). Previous studies demonstrated that PKC and MAPK signaling regulate gap-junction permeability, internalization, and degradation in astrocytes via C-terminal phosphorylation at Ser368 and MAPK-induced Ser279/Ser282 phosphorylation of Cx43 [46, 47], both of which we found to be induced by OXT. The transient recovery of Cx43 localization at cell/cell contacts after 30 min of OXT exposure might be due to the increase in Cx43 (*Gja1*) mRNA expression detected at this timepoint and might reflect a compensatory feedback mechanism. However, prolonged stimulation with OXT mimicked the effect of acute exposure on GJIC and was accompanied by decreased *Gja1* expression at this timepoint, indicating a manifestation of the inhibitory effect on gap-junctions, when astrocytes are exposed to OXT for longer periods of time. The fact that OXT induces a dynamic and isoform-specific expression of connexin-coding genes (Fig. S5) underlines its capacity to inhibit intercellular connectivity. Alterations in the interconnectivity of astroglial networks produce profound effects on synaptic transmission and plasticity on multiple levels. First, metabolites required at sites of high neuronal activity are partially trafficked via astroglial gap-junctions, which have been shown to undergo activity-dependent reshaping to meet this demand [48, 49]. Second, both potassium and glutamate reuptake efficiency are enhanced in areas of coupled astrocytes, pointing towards an inhibitory role of astroglial networks [50]. Last, gliotransmission induced by neuronal activity can additionally be triggered at distal synapses by information spread via astroglial networks and, thereby, lead to secondary activation of neurons [51–53]. In case of Cx30, it controls synapse invasion [54], synaptic glutamate clearance [54, 55] and cellular polarity [56]. Thus, the acute upregulation of astrocytic Cx30 by OXT could cause a less efficient glutamate uptake akin to the implications of OXT-induced EAAT2 downregulation. Our results demonstrate the regulation of astrocytic gap-junctions and their forming proteins by OXT, potentially facilitating excitatory transmission. However, the consequences on neuronal communication should be addressed by future work.

Since astrocytes modulate and support neuronal functions via facilitated diffusion of signaling molecules, ions or metabolites, the spatial arrangement and distance between astrocytic processes and neuronal structures are of functional importance [57]. This relationship is critically set by the astrocytic cytoskeleton [58, 59]. We revealed that OXT alters the expression of proteins associated with cytoskeletal dynamics, such as beta-tubulin, elements of the ROCK-pathway and GFAP (Fig. 1a). Consequently, we investigated OXT effects on astrocyte morphology and astrocyte-neuron spatial relationships. Indeed, OXTR mediates cytoskeletal remodeling in vitro and in vivo, conveyed via PKC and, to a lesser extent, MEK1/2 signaling. Specifically, we could show that OXT induces process elongation and ramification both in astrocytic cultures as well as within the rat PVN, leading to an increased astrocytic coverage of OXT neurons (Fig. 2). These results are generally supporting OXT as a regulator of cellular morphology in a cell type-dependent manner [41, 60–63]. Interestingly, it has repeatedly been observed that both hyperosmotic stress [64, 65] and elevated levels of OXT in the hypothalamic SON and PVN, e.g., during periods of heightened OXT activity such as lactation [17, 63], led to the retraction of astrocytic processes and increased neuronal-somata contacts, indicating OXT’s impact on the astrocyte-neuron spatial relationship [42]. However, the OXT-induced elongation and ramification of astrocytes we have observed in vitro and in male rats as well as the increased coverage of PVN OXT neurons following OXT-exposure (Fig. 2i) contradict the retraction of astroglial processes observed in the hypothalamic SON in lactation [63]. This discrepancy might be due to the variations in the mobility of major astrocytic branches, visualized by GFAP, compared to their fine, GFAP-negative, distal processes. To overcome this limitation, we expressed GFP in astrocytes followed by 3D-reconstruction and assessment of astrocytic synaptic coverage. We found a greater distance between synapses and GFP-labeled astrocytic elements in OXT-treated hippocampal slices, which is consistent with the decreased astroglial coverage described in the SON [63]. Notably, in the same acute slices, we could reproduce elongation and ramification of GFAP-positive branches, confirming the above described difference between major and fine branches. In the SON, the reduced astroglial synaptic coverage caused by OXT was reported to increase glutamate availability [19] and spillover to neighbouring synapses, facilitating excitatory transmission [20]. Importantly, the OXT-induced alterations in astrocytic morphology were highly specific as AVP, the related peptide had no effect, supporting findings in the SON [63].

Interestingly, recent studies also describe that OXT induces neurite retraction in hypothalamic H32 cells [41], influencing cytoskeletal proteins involved in neurite outgrowth [66, 67], which was shown to be mediated via inhibition of the calcium voltage-gated channels [68]. This aspect further underlines OXT´s potential impact on synaptic spatial relationships. The observed changes in these relationships may be due to a concerted, synergistic, effect of OXT on neuronal and astrocyte dynamics under the control of a differential regulation of the ROCK-pathway (Fig. 6).

ROCK signaling is involved in various cytoskeleton-associated cellular processes like contractility or migration, and OXT activates myometrial ROCK to increase uterine contractility [45]. Here, we describe that OXT effects on astrocytic cytoskeletal dynamics and GJIC are mediated by Gem, an endogenous inhibitor of the RhoA/ROCK pathway [69, 70]. Hallmarks of astrocytic RhoA/ROCK activation are retraction or loss of processes, whereas decreased RhoA/ROCK activity causes formation and outgrowth of processes, and breakdown of F-actin stress fibers [34–36], similar to the observed effects of OXT (Fig. 2c). In our astrocytic cultures, Gem prevented the OXT-induced activation of RhoA/ROCK (Fig. 3a, b) and, thus, seems central for intracellular OXT effects [45, 71]. Gem expression is facilitated by PKC [72–74], which aligns with the observed PKC dependency of OXT effects on astrocytic cytoskeletal parameters and suggests that OXT regulates Gem through PKC signaling. The unique characteristics of Gem and the dependence of OXT effects on Gem highlight it as a key factor in the cell type-specific response of astrocytes to OXT.

This is supported by the finding that Gem does not mediate OXT-induced cytoskeletal remodeling, but directly governed gap-junctional coupling, as well as Cx43 localization and phosphorylation status. The link between the cytoskeleton and GJIC is still under debate [75–77]. In cultured astrocytes, ROCK-inhibition alone did not reduce GJIC [78], but overexpression of Gem was sufficient to impair GJIC, suggesting that Gem controls Cx43 via inhibition of RhoA, but not ROCK. In support, direct activation of RhoA impaired GJIC in cardiac myocytes [77], and Gem inhibited RhoA independently of ROCK [69]. Our attempts to detect OXT-induced inhibition of RhoA activity by means of a pull-down assay of GTP-bound (i.e., active) RhoA failed due to low basal RhoA activity below detection limit; low astrocytic RhoA levels were also shown in the rat spinal cord [79]. Nevertheless, even low levels of Rho GTPase expression are physiologically relevant, as introduction of constitutively active or dominant-negative RhoA or Rac1 affected astrocyte morphology [36, 80].

To further strengthen the link between astrocytic gap-junction proteins and cytoskeletal plasticity, we used connexin knockout mice and found that Cx43, but not Cx30, is essential for OXT-induced cytoskeletal remodeling. Cx43 has been shown to play a central role in cytoskeletal dynamics in different cell types (reviewed in [81, 82]. Although Cx43 knockdown did not directly alter Gem, it was negatively correlated with the degree of active, i.e., phosphorylated Ezrin. pEzrin mediates Gem effects on cytoskeletal dynamics, as it interacts with Cx43, recruits Gem to the cell membrane and enables its inhibition of RhoA [69]. Adapter proteins like Ezrin, which are preferentially expressed in astrocytes [83], are part of Cx43 signaling scaffolds connecting Cx43 to the cytoskeleton [75, 84, 85], and may link the Gem and Cx43-mediated cytoskeletal dynamics elicited by OXT. The finding of PKC-induced phosphorylation of Ezrin [86] and the critical involvement of PKC-signaling in the astrocytic effects of OXT further support such a link. However, both Cx43 and Cx30 have been implicated in the regulation of cytoskeletal processes [56]. Although we found that Cx30 is not required for OXT-induced astrocytic remodeling in acute hippocampal slices, it is upregulated by OXT (Fig. 1a) and may compensate for the loss of Cx43-based gap-junctional coupling in vivo.

Taken together, OXT-induced cytoskeletal remodeling of astrocytes specifically involves the connexin isoform Cx43. This is likely mediated via C-terminal interactions of Cx43 that provide a link to the cytoskeleton and do not involve its channel function. Loss of Cx43 increases the available amount of active Ezrin, a protein required for the regulatory function of Gem on the cytoskeleton.

Investigating the underlying transcriptional mechanisms, we revealed that Sp1 drives OXT-induced expression of Gem in astrocytes. Sp1 is predominantly expressed by astrocytes [87, 88], and Sp1 knockout impairs neuronal outgrowth and alters astrocyte morphology accompanied by cognitive deficits [87]. Even though in our study knockdown of Sp1 did not produce noticeable effects (possibly due to residual Sp1 activity in the siRNA-transfected cells), OXT stimulation caused process retraction in Sp1 knockdown cells (Fig. 5b), similar to the effects in Gem knockdown cells (Fig. 3a, b). As OXT activated CREB in astrocytes (Fig. 1a), and Sp1 interacts with CREB among other transcription factors [89], *Gem* might also be regulated via such interaction. Thus, future work should validate direct binding of Sp1 to the promoter region of *Gem* by means of chromatin immunoprecipitation.

Interestingly, in contrast to astrocytes, OXT downregulated Sp1 and Gem in neurons, accompanied by an increase in ROCK activity, which we found to be necessary for neurite retraction in neuronal cultured cells. Such differential and even contrary responses of neurons and astrocytes have been repeatedly described [90–92]. In our in vivo experiments (Fig. 6), two-thirds of Gem-positive cells were of astrocytic identity, which is in accordance with its preferential astrocytic expression. The differential effects of OXT on neurons and astrocytes might induce a synergistic effect on synaptic spatial relationships; on one hand astrocytes are enlarged, on the other hand neuronal structures loosen their contacts to astrocytic elements by retraction.

Overall, we could link Sp1 to the regulation of Gem and to OXTR-coupled signaling. Thus, the previously undescribed OXTR-Sp1-Gem signaling axis could be identified as primary driver of cell type-specific OXT actions on astrocytes.

Finally, we extend the behavioral relevance of OXTR signaling to astrocytes using astrocyte-specific OXTR shRNA or Gem shRNA vectors. In rats expressing lower levels of either OXTR or Gem specifically in astrocytes of the PVN, the local anxiolytic effect of synthetic OXT confirmed in control rats both in the EPM and LDB [1, 10, 11] was abolished, which demonstrates that this effect of OXT in the hypothalamus requires direct binding to astrocytes. In support, astrocytic OXTR signalling in the rat central amygdala has recently been shown to be essential for OXT-induced reduction in pain-related anxiety [24].

In summary, by identifying astrocyte-specific signaling cascades, our study provides insights into the brain OXT system and the recruitment of astrocytes for the effects of OXT on cell morphology, cell inter-connectivity, and behaviour. While the involvement of neuronal OXTR-mediated signalling in anxiety-related and multiple other behaviors has been extensively studied [1, 2] the contribution of astrocytic OXT signaling has received limited attention until now [24]. Thus, our work provides basic information at molecular, cellular and behavioural levels for future investigations on the mechanisms of action of OXT.

## Supplementary information

FigS1

FigS2

FigS3

FigS4

FigS5

FigS6

FigS7

FigS8

Supplementary methods and supplemental figure legends

Supplementary tables
