# Supplementary figures and images for: OXTR-mediated signaling in astrocytes contributes to anxiolysis
Source: Mol Psychiatry. 2024 Dec 19;30(6):2620–34. doi: 10.1038/s41380-024-02870-5 (PMC12092269; doi:10.1038/s41380-024-02870-5)

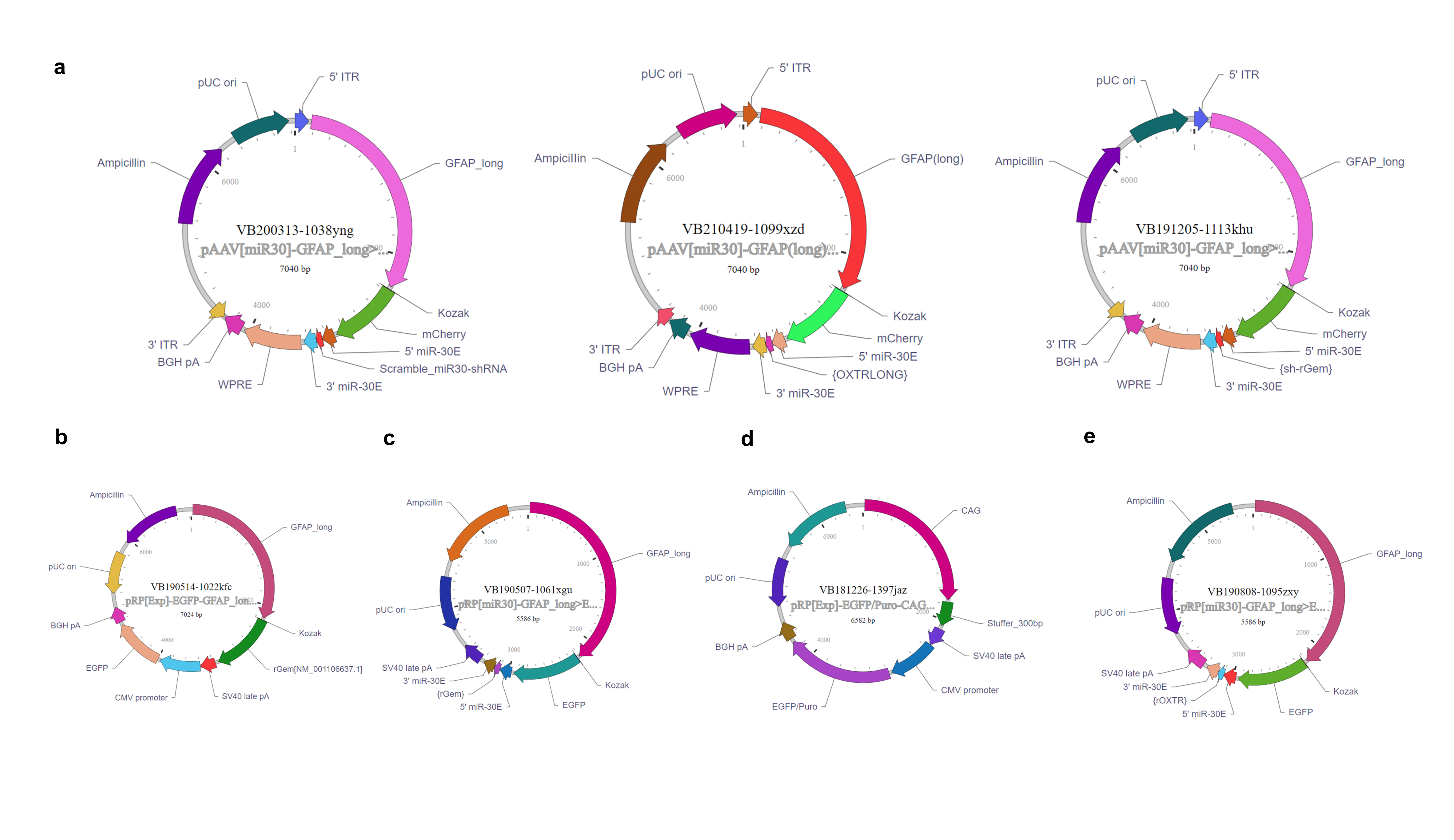

Supplement: Supplementary file 1 — FigS1 [file 41380_2024_2870_MOESM1_ESM.png]

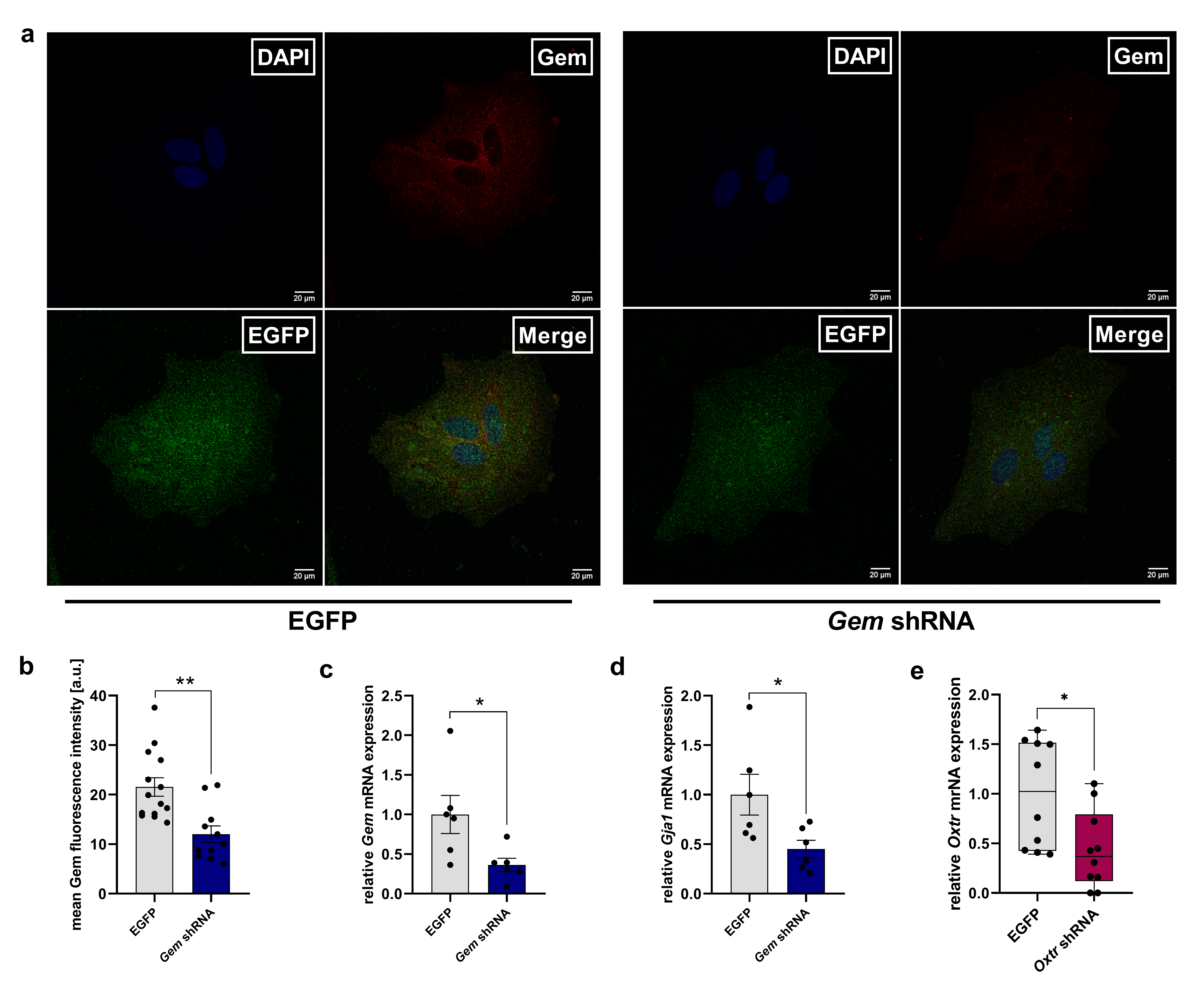

Supplement: Supplementary file 2 — FigS2 [file 41380_2024_2870_MOESM2_ESM.png]

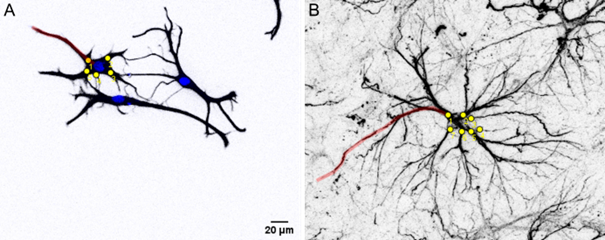

Supplement: Supplementary file 3 — FigS3 [file 41380_2024_2870_MOESM3_ESM.png]

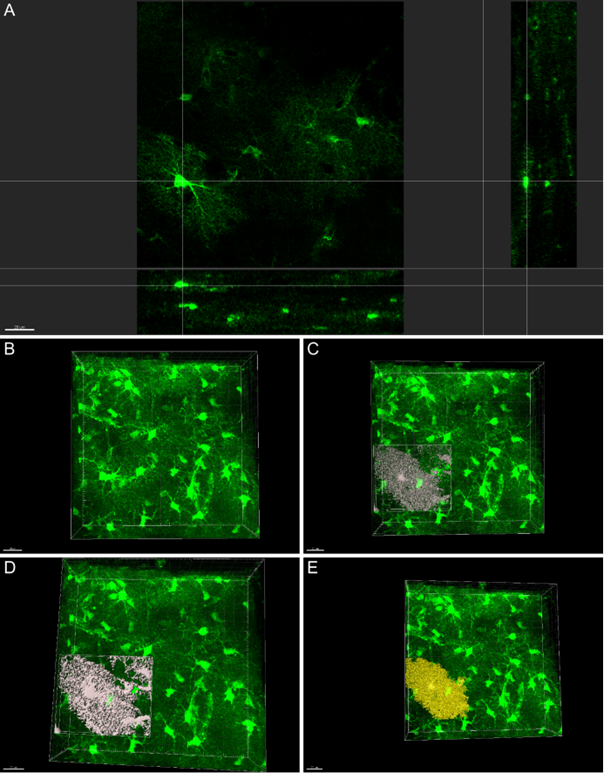

Supplement: Supplementary file 4 — FigS4 [file 41380_2024_2870_MOESM4_ESM.png]

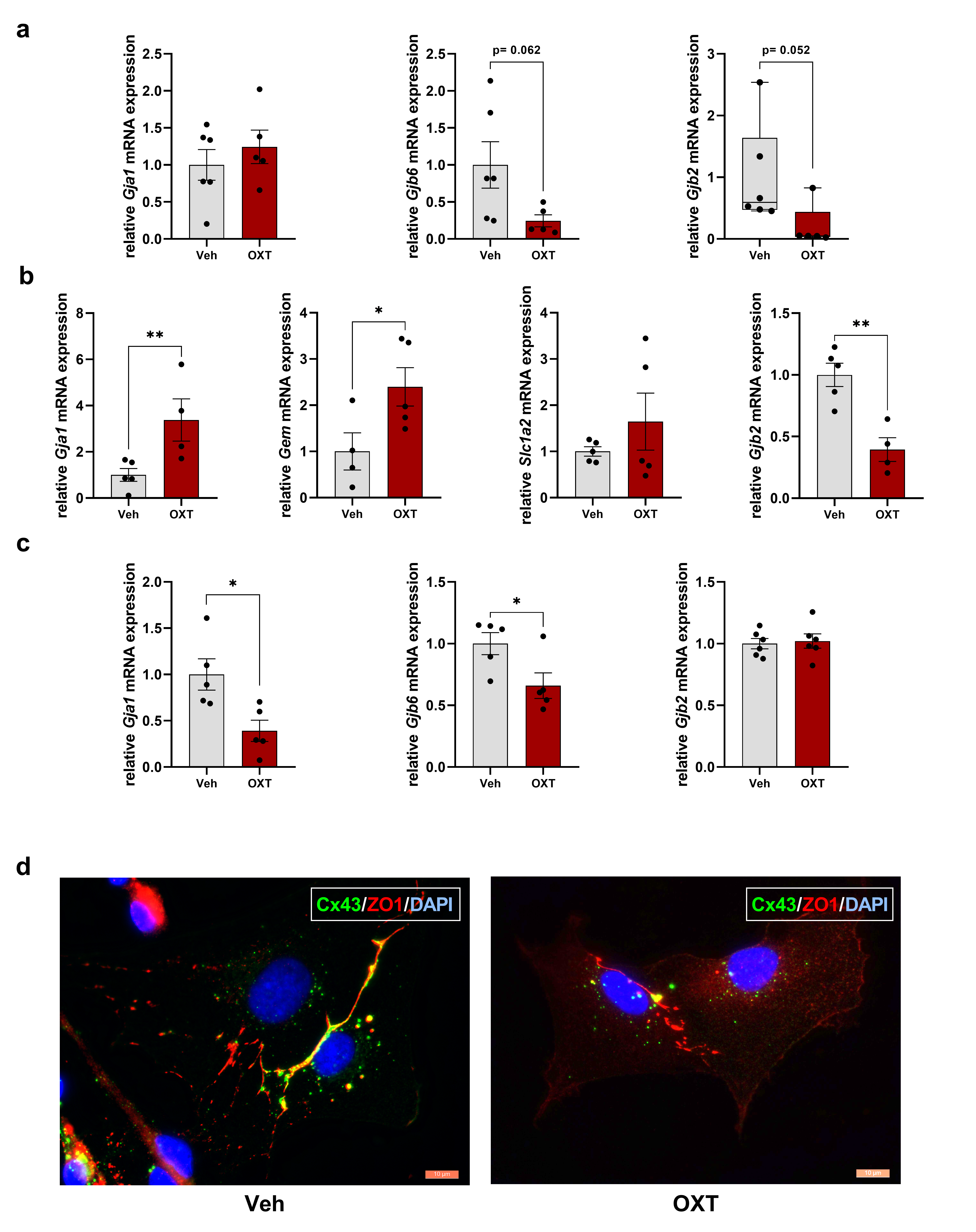

Supplement: Supplementary file 5 — FigS5 [file 41380_2024_2870_MOESM5_ESM.png]

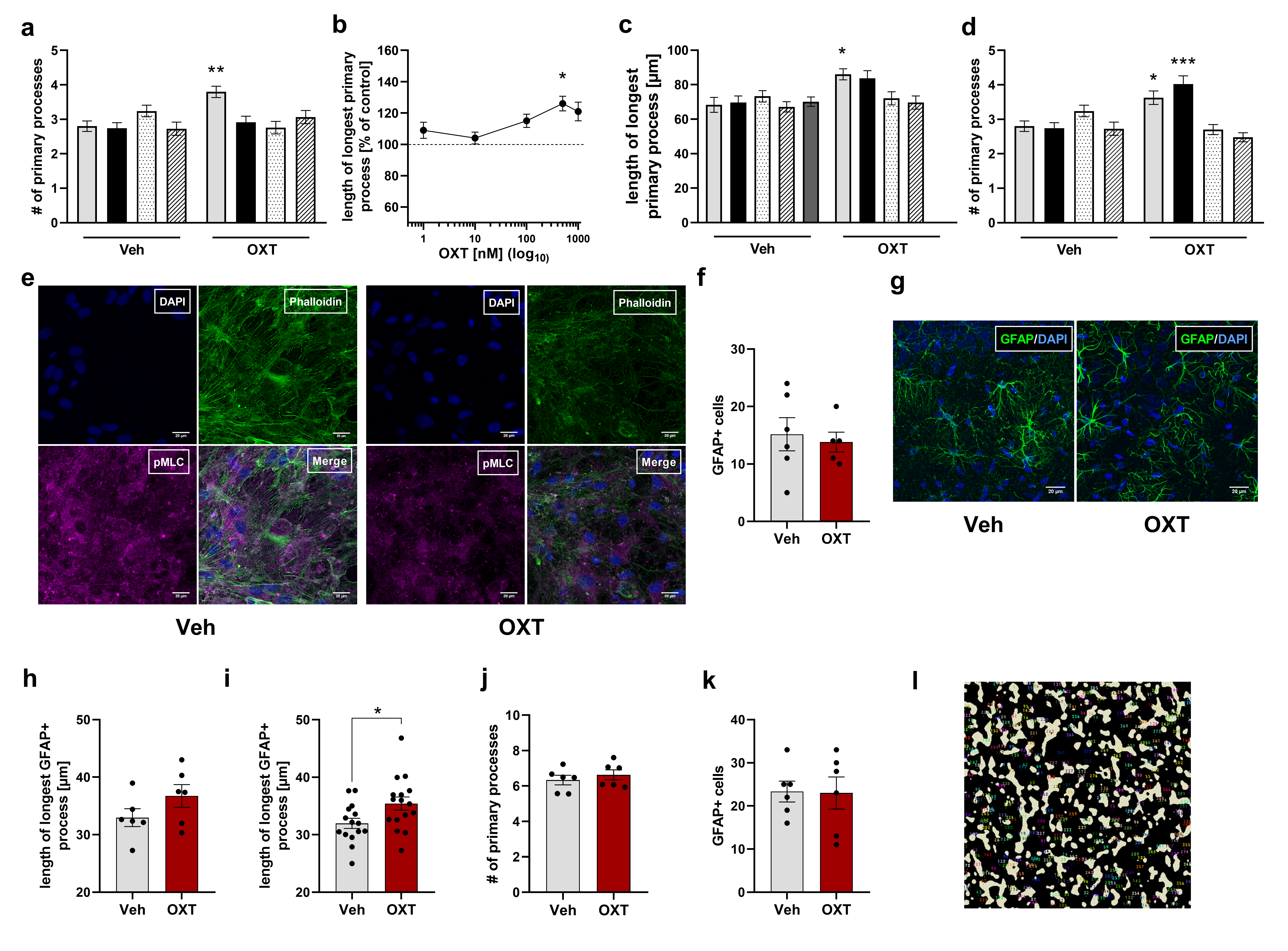

Supplement: Supplementary file 6 — FigS6 [file 41380_2024_2870_MOESM6_ESM.png]

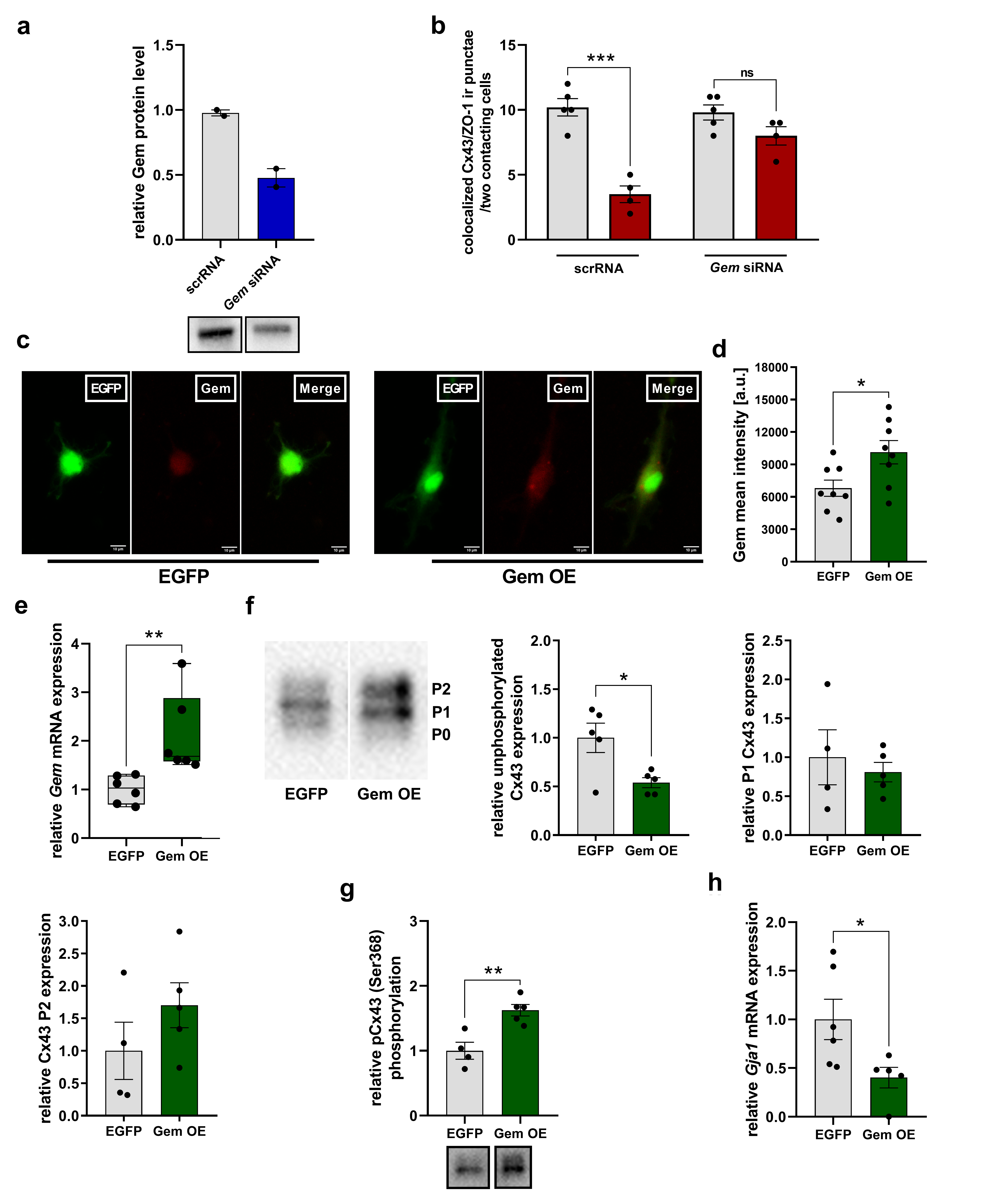

Supplement: Supplementary file 7 — FigS7 [file 41380_2024_2870_MOESM7_ESM.png]

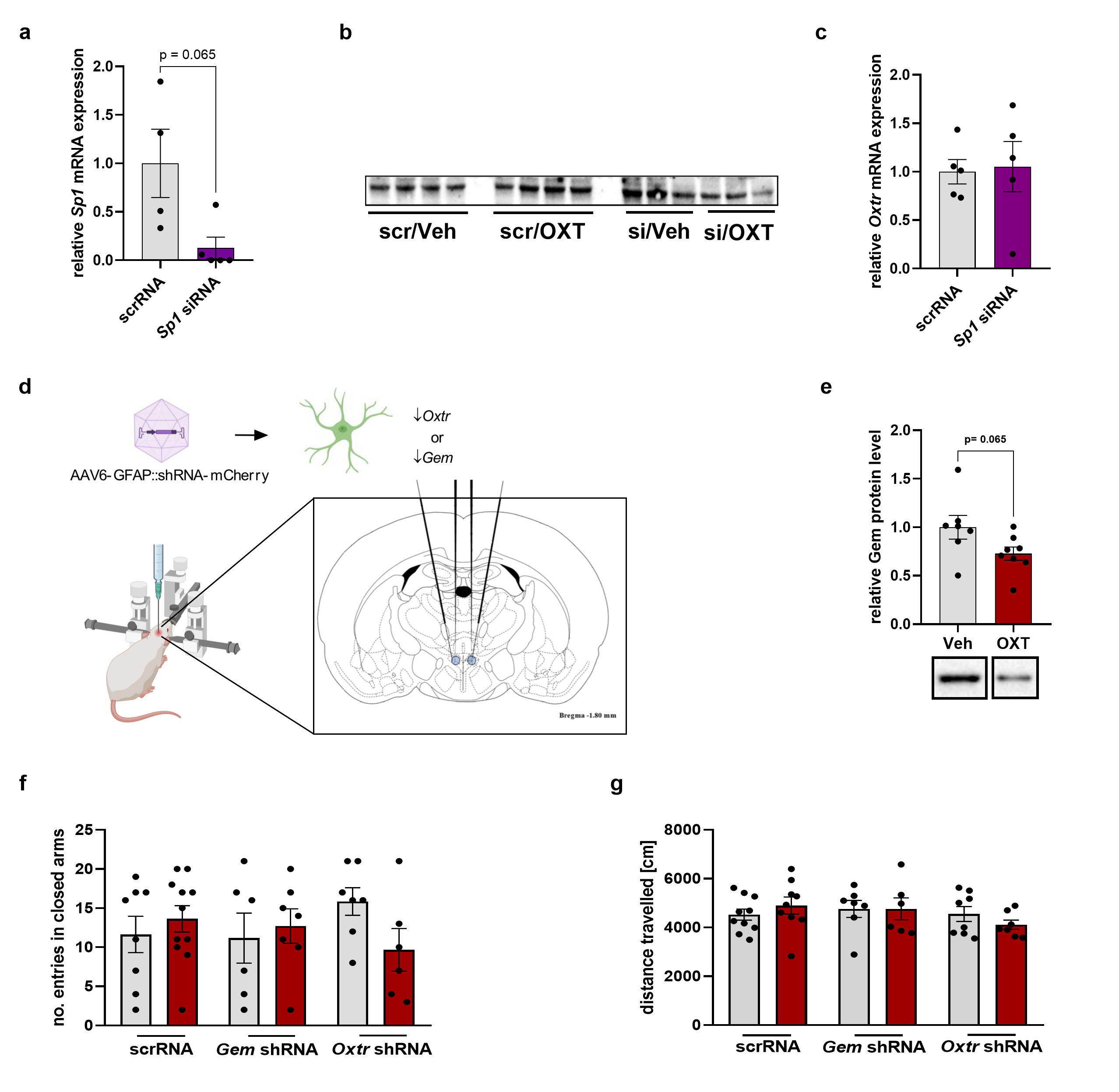

Supplement: Supplementary file 8 — FigS8 [file 41380_2024_2870_MOESM8_ESM.png]
