# Supplementary material for: OXTR-mediated signaling in astrocytes contributes to anxiolysis
Source: Mol Psychiatry. 2024 Dec 19;30(6):2620–34. doi: 10.1038/s41380-024-02870-5 (PMC12092269; doi:10.1038/s41380-024-02870-5)
Supplement: Supplementary file 9 — Supplementary methods and supplemental figure legends [file 41380_2024_2870_MOESM9_ESM.docx]

***Supplementary Methods***

***Guide cannula implantations***

Rats were anesthetized with isoflurane (Isofluran Baxter, Baxter Germany GmbH, Unterschleißheim, Germany). For *icv* or bilateral intra-PVN infusions, stainless steel guide cannulas (*icv*: 21G, 12mm long, x mm above the left lateral ventricle AP: -1.0mm bregma, ML: +1.6mm lateral, DV: +1.8mm;([G Paxinos, 2008](#_heading=h.3tbugp1)); for intra-PVN:: 23 G, 12 mm long, 2.0 mm above both the left and right PVN, AP: -1.4 mm bregma, ML: -1.8 mm and +/-2.1 mm lateral, DV: +6.0 mm, angle 10 ° Injecta GmbH, Klingenthal, Germany) were stereotaxically implanted and fixed to the skull with two stainless steel screws using dental cement. After surgery, an antibiotic (100µl, 2.5% Baytril®, Bayer Vital GmbH, Klingenthal, Germany) was administered subcutaneously to avoid post-surgical infections. Rats were handled daily for 5 days to reduce non-specific stress responses during experiments.

***Primary rat cortical astrocytes***

After resuspension in 10ml of astrocyte growth medium (Dulbecco’s Modified Eagle’s Medium (DMEM, high glucose), Sigma-Aldrich, St. Louis, USA; D6429) containing 10% fetal bovine serum (FBS), 1% penicillin/streptomycin (Life Technologies, Darmstadt, Germany), 1% MEM non-essential amino acid solution (100x, Sigma-Aldrich) and 1% Glutamax (Life Technologies; 35050038), the mixed cortical cells were seeded in poly-D-lysine coated (0.01% poly-D-lysine in H_2_O, Sigma Aldrich; P7886) T75 cell culture flasks (Sarstedt, Nürnbrecht, Germany). The flasks were incubated at 37°C and 5% CO_2_, and the medium was first changed after two days and subsequently every four days. After 7-8 days in culture, when the mixed cultures had reached confluence, the flasks were shaken on an orbital shaker for 30min at 37°C and 180rpm. After aspirating the supernatant containing microglial cells, fresh growth medium was added, and the cells were again shaken for 6h at 240 rpm to remove oligodendrocyte precursor cells. The remaining adherent astrocytic layer was trypsinated and seeded into two new TC75 cell culture flasks. After 7 or 14 days in culture, cells were seeded for experiments and treated as described below.

***Endpoint PCR and qPCR***

For endpoint PCR, 1µl cDNA, 2pmol of each forward and reverse primers (Metabion, Germany) and RNAse-free sterile H_2_O were added to DreamTaq™ Master Mix (Thermo Scientific). The PCR was run at the following thermal conditions: 95°C for 5min, followed by 40 cycles of 95°C for 15s and 72°C for 30s. PCR-products were visualized on a 1.5% agarose gel run at 140V for 45min using Roti®-Gel Stain (Carl Roth GmbH, Karlsruhe, Germany).

qPCR was performed with the QuantStudio 3 and QuantStudio 5 Real Time PCR Systems (ThermoFisher). One reaction mixture contained 5µl PowerUp™ SYBR® Green Master Mix (ThermoFisher; A25743), 9μl RNAse-free DEPC-treated H_2_O, 2μl of primers (4pmol) and 2µl cDNA reverse transcribed from 1µg RNA and diluted 1:2 in RNAse-free H_2_O. Reactions were run at the following thermal conditions: Uracil-DNA-Glycosylase activation at 50°C for 2min, hot-start activation of the Dual-Lock DNA polymerase at 95°C for 2min followed by 40 cycles of 95°C for 3s and 60°C for 30s. The housekeeping genes *Gapdh* and *Rpl* were used as internal reference controls and RNA expression was quantified by comparative _ΔΔ_ Ct-method. All primers pairs amplified a single product, as determined using a melt curve and additionally verified in an agarose gel electrophoresis.

***SDS-PAGE and Western Blotting***

20-30 μg of proteins were separated by SDS-PAGE on a 12.5% Criterion™ TGX Stain-Free™ Gel (Bio-Rad) for 20min at 70V followed by 2h at 100V. After crosslinking the trihalo components of the gel with tryptophan residues of the separated proteins in an UV-induced reaction, the proteins were transferred to a nitrocellulose membrane using the Trans-Blot Turbo System (Bio-Rad; 1704150). In order to visualize the total amount of blotted protein, the fluorescence of the crosslinked trihalo-tryptophan components was imaged at UV-light with the ChemiDoc XRS+ Imager (Bio-Rad). The picture of the total lane protein served as an internal reference control during the analysis. Blocking was carried out for 90min, followed by incubation with primary antibodies overnight (for antibodies and incubation conditions see Table S2). After incubation with respective secondary antibodies conjugated with horseradish peroxidase, the membranes were incubated for 5min with ECL developer solution (Bio-Rad; Table S2) and the protein/antibody complexes were then visualized with the ChemiDoc XRS+ Imager. The images were analyzed with ImageLab software (Bio-Rad) that was specifically created for the ChemiDoc Imager.

**Figure description**

**Figure S1.** Adenoviral vectors and DNA plasmids used. **a)** Vectors for astrocyte-specific knockdown of either *Oxtr* or *Gem* mRNA within the rat PVN, as well as control vector expressing scrRNA. **b-d)** DNA plasmids used for *in vitro* transfections. *Gem* overexpression plasmid expressing the *Gem* open reading frame (NCBI RefSeq NM_001106637.1) under the control of the long fragment of the *hGFAP* promoter and EGFP under the *CMV* promoter (B) and plasmids expressing a shRNA and EGFP under the control of the long fragment of the *hGFAP* promoter targeted against (C) the rat *Gem* mRNA or (D) the rat *Oxtr* mRNA. **e)** Control vector expressing solely EGFP under the control of the *CMV* promoter.

**Figure S2.** *In vitro* validation of shRNA-probes for knockdown of *Gem* or *Oxtr* mRNA in astrocytes. **a)** Representative images of primary astrocytes transfected with a control plasmid (left panel) or an shRNA targeted against the *Gem* mRNA. Scale bar = 20µm. **b/c)** Validation of successful Gem knockdown by quantification of immunofluorescence (B; t_23_=3.723, ** p=0.001) and qPCR (C; t_10_=3.506, p=0.031). **d)** Effect of Gem knockdown on *Gja1* mRNA levels (t_10_=2.443, * p=0.035). **e)** *Oxtr* mRNA levels analyzed by qPCR 7d after transfection of primary astrocytes with a plasmid expressing an shRNA targeted against *Oxtr* mRNA under the control of the long promoter fragment of the *hGFAP* gene (U=19, * p=0.018). Data represent mean relative or absolute values +/- SEM.

**Figure S3.** Quantification of length (red lines) and number (yellow dots) of primary GFAP+ processes of astrocytes *in vitro* and *in vivo* using ImageJ. **a)** Primary rat cortical astrocytes stained for GFAP/DAPI and analyzed for length of longest primary process as well as number of primary processes. **b)** Rat hippocampal (CA1 region) astrocyte stained for GFAP and analyzed for length of longest primary process, as well as number of primary processes. Lengths were measured from the edge of the nucleus indicated by DAPI staining to the end of the process of interest.

**Figure S4.** 3D reconstruction of GFP-expressing hippocampal mice astrocytes in acute *ex vivo* slice preparations using IMARIS. **a)** Astrocyte of interest depicted in a 2D image of the z-section. Lower/lateral panels show position of the cell in the context of the z-section. **b-c)** Generation of a 3D ROI around the cell of interest. **d-e)** Generation of a 3D object resembling the original shape of the astrocyte.

**Figure S5.** Expression of astrocytic genes following exposure to OXT for three differing timespans. **a)** *Gja1*, *Gjb6* and *Gjb2* mRNA levels after stimulation with 500nM OXT for 10min. **b)** *Gja1*, *Gem*, *Slc1a2* and *Gjb2* expression after 30 min of OXT exposure. **c)** *Gja1*, *Gjb6* and *Gjb2* mRNA levels following OXT application for 180min. **d)** Additional representative immunocytochemical images of astrocytes stained for Cx43 (green), the tight-junction protein ZO1 (red) and DAPI (blue) showing lower levels of Cx43/ZO1 colocalization in OXT- compared with Veh-treated cells..Data represent mean relative expression +/- SEM for normally distributed data and median +/- min/max values for non-normally distributed data. * p<0.05, ** p <0.01 (see Tab. S6 for statistical values).

**Figure S6.** Extended results of OXT effects on morphological parameters. **a)** Effect of 10min OXT exposure on the number of primary processes following pre-treatment with either Veh, U0126, Gö6983 or L368,889 (interaction F_3,414_=6.179, p=0.0004; ** p=0.002 for Veh/Veh vs. Veh/OXT. **b)** Same as Fig. 2B), but performed with 180min of OXT stimulation (F_5,554_=3.738, p=0.002; * p=0.016 for Veh/Veh vs. Veh/OXT). **c)** Same as Fig. 2C) but performed with 180min of OXT or AVP stimulation (interaction F_3,515_=2.800, p=0.04; * p=0.047 Veh/Veh vs Veh/OXT). **d)** Same as A) but performed with 180min of OXT stimulation (interaction F_3,450_=11.97, p<0.001; * p=0.018 Veh/Veh vs Veh/OXT; *** p<0.001 U0126/Veh vs. U0126/OXT). **e)** Representative staining of DAPI (blue), Phalloidin (green) and pMLC/Ser19 (magenta) in astrocytes treated with either Veh (left panel) or 500nM OXT for 3h (right panel). **f**) Number of GFAP+ cells within the rat PVN 10min after OXT administration. Scale bar = 30µm. **g)** ImageJ analysis plugin output displaying astrocytic elements in white and functional synapses as single-colored dots. Data represent mean +/- SEM.

**Figure S7.** Manipulation of Gem expression impacts phosphorylation and localization of Cx43. **a)** Validation of Gem knockdown by siRNA by immunoblotting. Representative bands are shown below. **b)** Cx43/Zo1-colocalization following transfection with either Gem siRNA or a control oligonucleotide and subsequent OXT stimulation for 15min (interaction: F_1,14_=14.04, p=0.002; *** p<0.001 scrRNA/Veh vs. scrRNA/OXT and p=0.4316 siRNA/Veh vs. siRNA/OXT). **c)** Representative images (EGFP, green; Gem, red) of cells transfected with an EGFP expressing control plasmid (left panel) or the ORF of the rat *Gem* mRNA (right panel) under the promoter of the *hGFAP* gene. **d)** Intensity of Gem-immunofluorescence in Gem OE cells (t_14_=2.539, * p=0.024). **e)** Validation of Gem OE on mRNA level (*U*=0, ** p = 0.002). **f)** Effect of Gem OE on the phosphorylation status of Cx43 with P0 representing the unphosphorylated form of Cx43 and P1/P2 representing two distinct phosphorylation sites (t_8_=2876, * p=0.021). **g-h)** Impact of Gem OE on pCx43(Ser368) phosphorylation levels (G; t_7_=4.090, ** p=0.005), as well as Cx43 (*Gja1*) mRNA (H; t_9_=2.414, * p=0.039).

**Figure S8.** The role of the transcription factor Sp1 in OXT-induced Gem expression (**a–c**) and experimental details on the involvement of astrocytic OXTR signaling on the locomotion effect of OXT within the PVN (**f-g**). **a)** Validation of successful Sp1 knockdown by qPCR (U=2, p=0.065). **b)** Representative immunoblot of Gem showing noticeable OXT-induced increase of intensity solely in the scrRNA group. **c)** Effects of Sp1 knockdown on *Oxtr* mRNA expression. **d)** Stereotactic coordinates for both placement of guide cannulas (angled thick outer line) and infusion cannulas (angled thin outer line) for local administration of OXT to the PVN. Inner triangular black lines represent glass cannula placement for AAV microinfusions. Torquoise circles mark points of infusions. Illustration adopted from Paxinos and Watson (2006). **e)** Effect of 10 min of exposure to OXT (100nM) on the protein level of Gem (t_13_=2.015, p=0.065) in H32 neurons assessed by immunoblotting. Representative bands are shown below. **f)** As indication of locomotor activity, the number of entries into closed arms of EPM (**f**) and distance travelled in the OF (**g**) are presented. Rats are pre-treated with either viral vectors for astrocyte-specific knockdown of OXTR (*Oxtr* shRNA) or Gem (*Gem* shRNA), or scrRNA 21d prior to behavioral testing and receiving intra-PVN microinfusions of either Veh or OXT 10 min prior to behavioral testing (see legend to Fig.5h). n=6-11/group. Data represent mean +/- SEM.
