# Supplementary material for: OXTR-mediated signaling in astrocytes contributes to anxiolysis
Source: Mol Psychiatry. 2024 Dec 19;30(6):2620–34. doi: 10.1038/s41380-024-02870-5 (PMC12092269; doi:10.1038/s41380-024-02870-5)
Supplement: Supplementary file 10 — Supplementary tables [file 41380_2024_2870_MOESM10_ESM.docx]

***Supplementary tables***

**Supplementary Table 1**. Primers with their respective PCR product size used in PCR and qPCR experiments.

| **Target** | **Forward primer (5’-3’)** | **Reverse primer (5’-3’)** | **Product size (bp)** |
| --- | --- | --- | --- |
| *Gem* | TGTGTCAGAAGGGAGAGCTTG | CAAGGGGACATCTGGACGAC | 315 |
| *Oxtr* | CTGGAGTGTCGAGTT GGACC | AGCCAGGAACAGAAT GAGGC | 136 |
| *Gja1* | TTCATTGGGGGAAAGGCGTG | CTGGGCACCTCTCTTTCACTT | 173 |
| *Gjb6* | TTCCAGTTCACCTCACACGG | GGCAGTGGGAATGTCACCTTT | 99 |
| *Gjb2* | GGAACGAGACTCAGGAGCGT | CGGGGAAGAAGTGGTCGTAG | 236 |
| *Slc1a2* | GTGGACTGGCTGCTGGATAG | AGTTGTGTGCGGCATAGACA | 223 |
| *Sp1* | AAACACCCCAGGTGATCATGG | CATGAATGGCCTCTCCCCTG | 307 |
| *Gapdh* | TGATGACATCAAGAA GGTGG | CATTGTCATACCAGG AAA TGAG | 185 |
| *Rpl* | ACAAGAAAAAGCGGA TGGTG | TTCCGGTAATGGATC TTTGC | 172 |

**Supplementary Table 2**. List of primary antibodies with their respective dilutions and secondary antibodies used in immunocytochemistry experiments.

| **Primary antibody** |  | **Secondary antibody**  (All ThermoFisher) |
| --- | --- | --- |
| GFAP cs12389  1:1000 |  | goat-anti rabbit AlexaFluor488  1:1000 |
| ZO1 ThermoFisher  1:100 |  | goat anti-mouse AlexaFluor594  1:1000 |
| Gem A-3 sc-514497  1:100 |  | goat anti-mouse AlexaFluor594  1:1000 |
| pMLC/Ser19 cs3671  1:50 |  | goat anti-rabbit AlexaFluor594  1:1000 |
| GFP ThermoFisher PA1-980A  1:200 |  | goat-anti rabbit AlexaFluor488  1:1000 |
| AlexaFluor488 Phalloidin cs8878  1:20 |  | - |
| AlexaFluor594 Phalloidin cs8953  1:20 |  | - |
|  |  |  |

**Supplementary Table 3**. List of primary antibodies with their respective dilutions and secondary antibodies used in immunohistochemistry experiments.

| **Primary antibody** |  | **Secondary antibody**  (All ThermoFisher) |
| --- | --- | --- |
| GFAP cs12389  1:500 |  | goat-anti rabbit AlexaFluor488  1:1000 |
| GFAP ab50738  1:500 |  | goat-anti chicken AlexaFluor488  1:1000 |
| Gem A-3 sc-514497  1:100 |  | goat anti-mouse AlexaFluor594  1:1000 |
| mCherry abcam 167453  1:800 |  | goat anti-rabbit AlexaFluor594  1:1000 |
| Homer1 SySy 160 003  1:250 |  | donkey anti-rabbit AlexaFluor647  1:500 |
| Vglut1 Sysy 135 311  1:250 |  | goat anti-mouse AlexaFluor594  1:500 |
| GFP Aves AB_2307313  1:500 |  | goat-anti chicken AlexaFluor488  1:500 |
| OXT-Neurophysin clone PS38 kindly provided by Dr. Harold Gainer  1:500 |  | goat anti-mouse AlexaFluor555  1:1000 |

**Supplementary Table 4.** Direction of statistically significant changes in protein/phosphorylation levels following exposure of primary rat cortical astrocytes to 500nM OXT for 10min or 10min/20min after *icv* administration of OXT in punches derived from three different brain regions (PVN, hippocampus, amygdala). Up = statistically significant upregulation; Down = statistically significant downregulation; - = assessed, but no significant change; n.a. = not assessed due to non-astrocyte-specific expression in vivo or lack of expression in vitro.

| **Target** | ***In vitro* change** | ***In vivo* change**  **(10 Min)** | ***In vivo* change**  **(20 Min)** | **Statistics** |
| --- | --- | --- | --- | --- |
| ***Cellular signaling*** | | | | |
| pCreb/Ser133 | Up | n.a. | n.a. | t15=2.840, p=0.012 |
| pAkt/Thr308 | Up | n.a. | n.a. | t15=2.303, p=0.036 |
| pERK1 | Up | n.a. | n.a. | t10=2.459, p=0.038 |
| pERK2 | Up | n.a. | n.a. | t10=4.702, p<0.001 pERK2 |
| pERK5/Thr218/Tyr220 | Up | n.a. | n.a. | t7=2.309, p=0.054 |
| pAMPK/Thr172 | - | n.a. | n.a. |  |
| pp38 | - | n.a. | n.a. |  |
| pJNK/Thr183/Thr185 | - | n.a. | n.a. |  |
| pcamKII/Thr286 | - | n.a. | n.a. |  |
| peEF/Thr56 | Down | n.a. | n.a. | U=0, p=0.008 |
| Sp1 | Up | n.a. | n.a. | t12=2.470, p=0.030 |
| ***Gap-junction proteins*** | | | | |
| pCx43/Ser368 | Up | Up (PVN/Hippcoampus) | Up (PVN) | t14=3.506 p=0.004 (in vitro), t11=2.244, p=0.046 (PVN 10min), t11=2.029, p=0.067 (PVN 20min), t10=2.195, p=0.053 (Hippocampus 10min) |
| pCx43/Ser279 | Up | n.a. | n.a. | U=1, p=0.016 |
| pCx43/P1 | Up | n.a. | n.a. | U=10, p=0.021 |
| pCx43/P2 | Up | n.a. | n.a. | t15=2.574 p=0.021 |
| Cx30 | n.a. | Up (PVN/Hippocampus/Amgydala) | Up (PVN) | t11=3.361, p=0.006 (PVN 10min); U=2, p=0.005 (PVN 20min), U=4, p=0.014 (Hippocampus 10min), U=7, p=0.051 (Amgydala 10min) |
| Cx43 | - | Down (PVN/Hippocampus) | Down (Hippocampus) | t11=3.546, p=0.005 (PVN 10min), t12=6.664, p<0.001 (Hippocampus 10min), t12=2.707, p=0.019 (Hippocampus 20min) |
| ***Cytoskeleton-related*** | | | | |
| pEzrin/Thr567 | Up | n.a. | n.a. | t10=2.536, p=0.030 |
| pMYPT/Thr696 | Down | n.a. | n.a. | t15=2.068, p=0.056 |
| ROCK1 | Up | n.a. | n.a. | t7=2.758, p=0.028 |
| Gem | Up | Up/Down (PVN/Amygdala) | Up (PVN) | t18=2.203, p=0.041 (In vitro), U=2, p=0.005 (PVN 10min), U=7, p=0.051 (PVN 20min), t12=2.818, p=0.016 (Amygdala 20min) |
| GFAP | Down | - | Up (Amygdala) | t8=2.718, p=0.026 (in vitro), U=4, p=0.014 (Amygdala 20min) |
| beta-Tubulin | Up | n.a. | n.a. | U=2, p=0.032 |
| ***Other astrocyte-specific*** | | | | |
| EAAT1 | n.a. | Up (Amygdala) | Down (Hippocampus) | t10=2.972, p=0.014 (Hippocampus 20min), t11=4.387, p=0.001 (Amygdala 10min) |
| EAAT2 | - | Down (PVN) | Down (Amygdala) | t12=2.799, p=0.016 (PVN 10min), t12=2.533, p=0.026 (Amygdala 20min) |

**Supplementary Table 5.** Direction of expression of astrocytic genes following exposure to OXT for three different timespans. - = assessed, but no significant change.

| **Target** | ***Direction of change*** | **Statistics** |
| --- | --- | --- |
| ***10min post-administration*** | | |
| Cx43 (*Gja1*) | - |  |
| Cx30 (*Gjb6*) | Down | t9=2.134, p=0.062 |
| Cx26 (*Gjb2*) | Down | *U*=4, p=0.052 |
| ***30min post-administration*** | | |
| Cx43 (*Gja1*) | Up | t_7_=2.755, p=0.028 |
| Cx26 (*Gjb2*) | Down | t_7_=4.427, p=0.003 |
| *Gem* | Up | t_7_=2.373, p=0.049 |
| *Slc1a2* | - |  |
| ***180min post-administration*** | | |
| Cx43 (*Gja1*) | Down | t_8_=2.982, p=0.018 |
| Cx30 (*Gjb6*) | Down | t_8_=2.486, p=0.039 |
| Cx26 (*Gjb2*) | - |  |

|  |  |  |
| --- | --- | --- |

**Supplementary Table 6**. List of predicted transcription factor binding sites within the promoter region of the rat *Gem* gene.

| **Transcription factor** |  | **# of predicted binding sites within the promoter region of *Gem*** |
| --- | --- | --- |
| Sp1 |  | 12 |
| NF-kappaB |  | 2 |
| AP-2alpha |  | 2 |
| AP-1 |  | 1 |
| HNF-1C |  | 1 |
| CEPB-alpha |  | 1 |
| CEPB-beta |  | 1 |
| Pit-1b |  | 1 |
| NF-1 |  | 1 |
| ETF |  | 1 |
| WT1 |  | 1 |
| c-Fos |  | 1 |
| CREB |  | 1 |
| ATF |  | 1 |
| E1A 12S |  | 1 |
| RxR-beta |  | 1 |
| c-Jun |  | 1 |
| NF-kappa |  | 1 |
| CRE-BP1 |  | 1 |

**Supplementary Table 7**. List of antibodies used in immunoblotting experiments with their respective application protocols.

| **Primary antibody** | **Secondary antibody** | **Blotting protocol** | **Blocking solution** | **Developer solution** |
| --- | --- | --- | --- | --- |
| pCREB (Ser133) Milipore 06519  1:5000 in 5% BSA | Anti-rabbit lgG, 7074S  1:5000 in 5% BSA | StandardSD (30min) | 5% BSA | Clarity™ Western ECL Substrate (Bio-Rad) |
| pCamKII (Thr286) cs12716S  1:1000 in 5% BSA | Anti-rabbit lgG, 7074S  1:1000 in 5% BSA | StandardSD (30min) | 5% BSA | Clarity™ Western ECL Substrate |
| Sp1 Milipore 07645  1:1000 in 5% BSA | Anti-rabbit lgG, 7074S  1:5000 in 5% MP | StandardSD (30min) | 5% BSA | Clarity™ Western ECL Substrate |
| Cx43 cs3512S  1:1000 in 5% BSA | Anti-rabbit lgG, 7074S  1:1000 in TBS-T | StandardSD  (30min) | 5% BSA | Clarity™ Western ECL Substrate |
| pCx43 (Ser282) Thermo PA5-64641  1:500 in 5% BSA | Anti-rabbit lgG, 7074S  1:1000 in TBS-T | StandardSD (30min) | 5% BSA | Super Signal^TM^ West Dura Extended Duration Substrate (ThermoFisher) |
| pCx43 (Ser279) Thermo PA5-64777  1:500 in 5% BSA | Anti-rabbit lgG, 7074S  1:2000 in 2% BSA | StandardSD (30min) | 5% BSA | Clarity Max™ Western ECL Substrate (Bio-Rad) |
| pCx43 (Ser368) cs3511S  1:1000 in 5% BSA | Anti-rabbit lgG, 7074S  1:1000 in TBS-T | StandardSD (30min) | 5% BSA | Clarity™ Western ECL Substrate |
| Cx30 Thermo 71-2200 1:250 in 5% MP  Samples heated to 70°C for 15min | Anti-rabbit lgG, 7074S  1:1000 in TBS-T | StandardSD (30min) | 5% MP | Super Signal^TM^ West Dura Extended Duration Substrate |
| MYPT1 cs2634S  1:1000 in 5% BSA | Anti-rabbit lgG, 7074S  1:1000 in TBS-T | StandardSD (30min) | 5% BSA | Clarity Max™ Western ECL Substrate |
| pMYPT1 (Thr696) cs4563S  1:500 in 5% MP | Anti-rabbit lgG, 7074S  1:1000 in 3% MP | StandardSD (30min) | 5% MP | Clarity Max™ Western ECL Substrate |
| pSAPK/JNK (Thr183/Tyr185) cs9251S  1:5000 in 5% BSA | Anti-rabbit lgG, 7074S  1:5000 in TBS-T | StandardSD (30min) | 5% BSA | Super Signal^TM^ West Dura Extended Duration Substrate |
| pAkt (Thr308) cs13038S  1:1000 in 5% MP | Anti-rabbit lgG, 7074S  1:5000 in 5% MP | StandardSD (30min) | 5% MP | Clarity Max™ Western ECL Substrate |
| Gem A-3 sc-514497  1:2000 in 5% BSA | m-lgGκ BP-HRP sc-516102  1:2000 in TBS-T | Low MW (5min) | 5% BSA | Clarity Max™ Western ECL Substrate |
| peEF2 (Thr56) cs2331  1:000 in 5% BSA | Anti-rabbit lgG, 7074S  1:1000 in TBS-T | StandardSD (30min) | 5% MP | Clarity™ Western ECL Substrate |
| ROCK1 cs4035  1:2000 in 5% BSA | Anti-rabbit lgG, 7074S  1:2000 in TBS-T | StandardSD (30min) | 5% BSA | Super Signal^TM^ West Dura Extended Duration Substrate |
| pAMPK (Thr172) cs2535  1:2000 in 5% BSA | Anti-rabbit lgG, 7074S  1:2000 in TBS-T | StandardSD (30min) | 5% BSA | Clarity™ Western ECL Substrate |
| EAAT1 sc515839  1:400 in 5% BSA | m-lgGκ BP-HRP sc-516102  1:1000 in TBS-T | StandardSD (30min) | 5% BSA | Super Signal^TM^ West Dura Extended Duration Substrate |
| EAAT2 sc365634  1:10000 in 5% BSA | m-lgGκ BP-HRP sc-516102  1:2000 in TBS-T | StandardSD (30min) | 5% BSA | Clarity™ Western ECL Substrate |
| pp38 (Thr180/Tyr182) sc17852  1:1000 in 5% BSA | Anti-rabbit lgG, 7074S  1:1000 in TBS-T | Mixed MW (7min) | 5% BSA | Clarity™ Western ECL Substrate |
| pERK1/2 cs9101  1:5000 in 5% BSA | Anti-rabbit lgG, 7074S  1:1000 in TBS-T | StandardSD (30min) | 5% BSA | Clarity™ Western ECL Substrate |
| ERK1/2 cs9102  1:1000 in 5% BSA | Anti-rabbit lgG, 7074S  1:1000 in TBS-T | StandardSD (30min) | 5% BSA | Clarity™ Western ECL Substrate |
| pERK5 (Thr218/Tyr220) Millipore 07-507  1:5000 in 3% MP | Anti-rabbit lgG, 7074S  1:5000 in 3% MP | StandardSD (30min) | 3% MP | Clarity Max™ Western ECL Substrate |
| GFAP cs12389  1:5000 in 5% BSA | Anti-rabbit lgG, 7074S  1:2000 in TBS-T | StandardSD (30min) | 5% BSA | Clarity™ Western ECL Substrate |
| Beta-Tubulin cs2146  1:1000 in 5% MP | Anti-rabbit lgG, 7074S  1:1000 in TBS-T | StandardSD (30min) | 5% MP | Clarity™ Western ECL Substrate |
| pEzrin (Thr567) ab47293  1:1000 in 5%BSA | Anti-rabbit lgG,  1:1000 in 5%BSA | StandardSD (30min) | 5% BSA | Super Signal^TM^ West Dura Extended Duration Substrate |
